# Supplementary material for: Genomic instability and biofilm determinants in Streptococcus mutans: insights from a sequence-defined arrayed transposon library
Source: J Bacteriol. 2026 Jun 22;208(7):e00174-26. doi: 10.1128/jb.00174-26 (PMC13393454; doi:10.1128/jb.00174-26)
Supplement: Supplemental materials — Figures S1 to S22, Tables S2 to S6, and scripts. [file jb.00174-26-s0001.pdf]

Supplemental Material for:

Title: Genomic instability and biofilm determinants in *Streptococcus mutans*: insights from a sequence-defined arrayed transposon library

Authors: Ana Karen Solano Morales[1,2], Emanuel Cazano[1], Cara Pirani[1], Graysen Jones[1], Andrew Goode[1], Alejandro R. Walker[2], Anthony Sperduto[2], Bikash Dwivedi[2], Pallavi Bantha[3], Stella D. Peter[4], Lisa K. McLellan[4], Mohamed A. Alam[3] and Robert C. Shields[1,2]

Affiliations:
[1]Department of Biological Sciences, Beck College of Sciences and Mathematics, Arkansas State University, Jonesboro, Arkansas, USA
[2]Department of Oral Biology, College of Dentistry, University of Florida, Gainesville, Florida, USA
[3]Department of Chemistry, Beck College of Sciences and Mathematics, Arkansas State University, Jonesboro, Arkansas, USA
[4]Department of Biological Sciences, College of Science, Purdue University Fort Wayne, Fort Wayne, Indiana, USA

*Address correspondence to: rshields@dental.ufl.edu

This file includes:
Figures S1 - S22
Tables S2-S6
Scripts
- Linux command line
- Rstudio

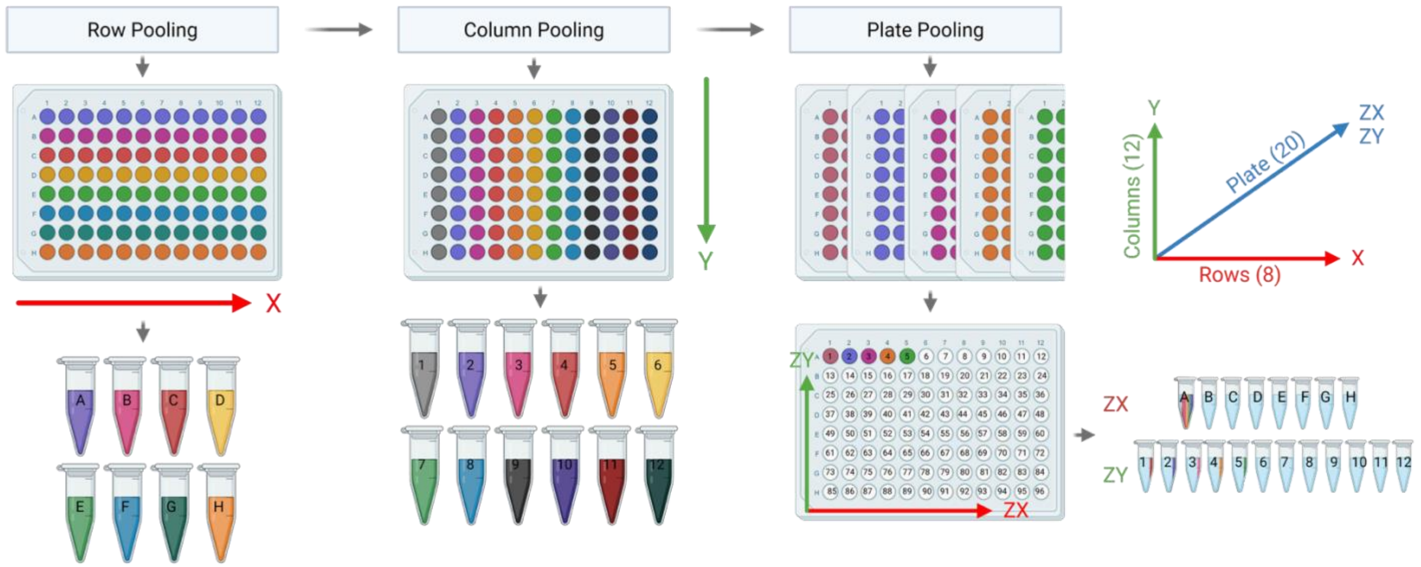

Figure S1 Cartesian Pooling-Coordinate Sequencing (CP-CSeq) strategy for mutant deconvolution. The pooling scheme utilized a three-dimensional coordinate system to uniquely identify the plate position of transposon mutants within the 9,216-well library. Genomic DNA was pooled across three axes: (X) row-pools (8 total; A-H), (Y) column-pools (12 total; 1-12), and (Z) plate-pools (20 total; ZX and ZY), resulting in a total of 40 distinct combinatorial pools. Each individual mutant is defined by a unique "coordinate address" composed of its presence in exactly one row-pool, one column-pool, and two plate-specific pools (Z-coordinate). For sequencing, 20 barcodes were used, and the 40 pools were therefore processed into two sets of 20. The first set contained the row and column pools (X and Y), and the second set contained the plate pools (ZX and ZY). During bioinformatic deconvolution, the overlap of sequencing reads across these axes allows the genomic insertion site identified by NovaSeq to be mapped back to a specific well in the physical library. The plate pooling (ZX and ZY) is used to determine which plate a mutant resides in. To identify the well within a plate that the mutant resides in, the row and column pools (X and Y) are used.

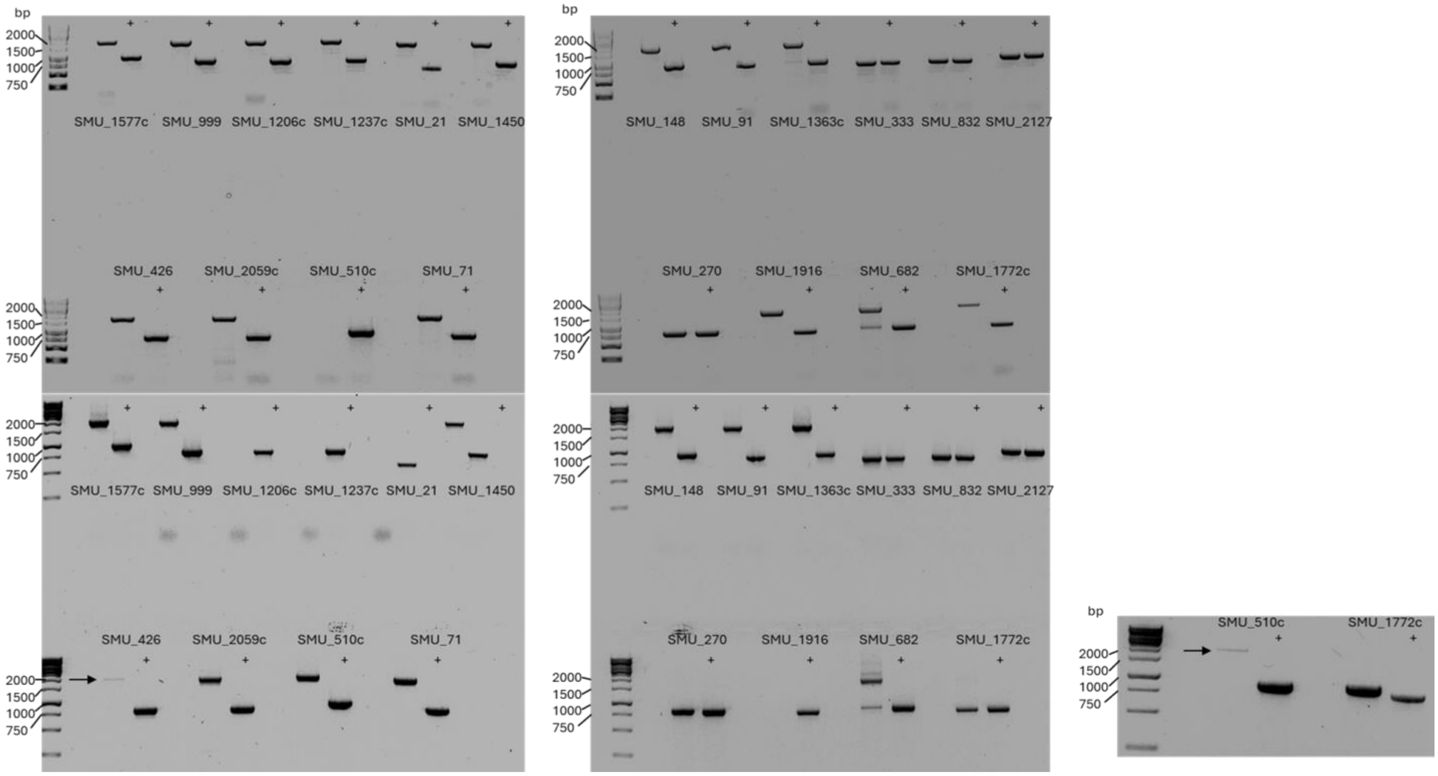

Figure S2 PCR verification of twenty CP-CSeq assigned *S. mutans* transposon mutants. A 1.8% agarose gel was used to run the PCR verification products generated for twenty randomly selected mutants from the arrayed *S. mutans* UA159 transposon library. Primers flanking each CP-CSeq-predicted insertion site were used to detect the presence of the transposon disrupted allele. The (+) lane corresponds to the positive control (*S. mutans* wild type) amplified with the same gene-specific primers. Because the wild-type allele contains the intact gene sequence and the mutant allele contains a ~1.1-kb transposon insertion between the primer bindings sites, the PCR product bands appear larger than the (+) control band. Successful amplification of the expected product in mutant lanes indicates concordance between the bioinformatically assigned insertion site and the observed PCR band. A total of 16 out of 20 mutants (80%) produced amplicons of the expected size, confirming accurate CP-CSeq deconvolution, whereas four mutants failed to amplify. A 1 kb DNA ladder is shown in the first lane of each gel panel.

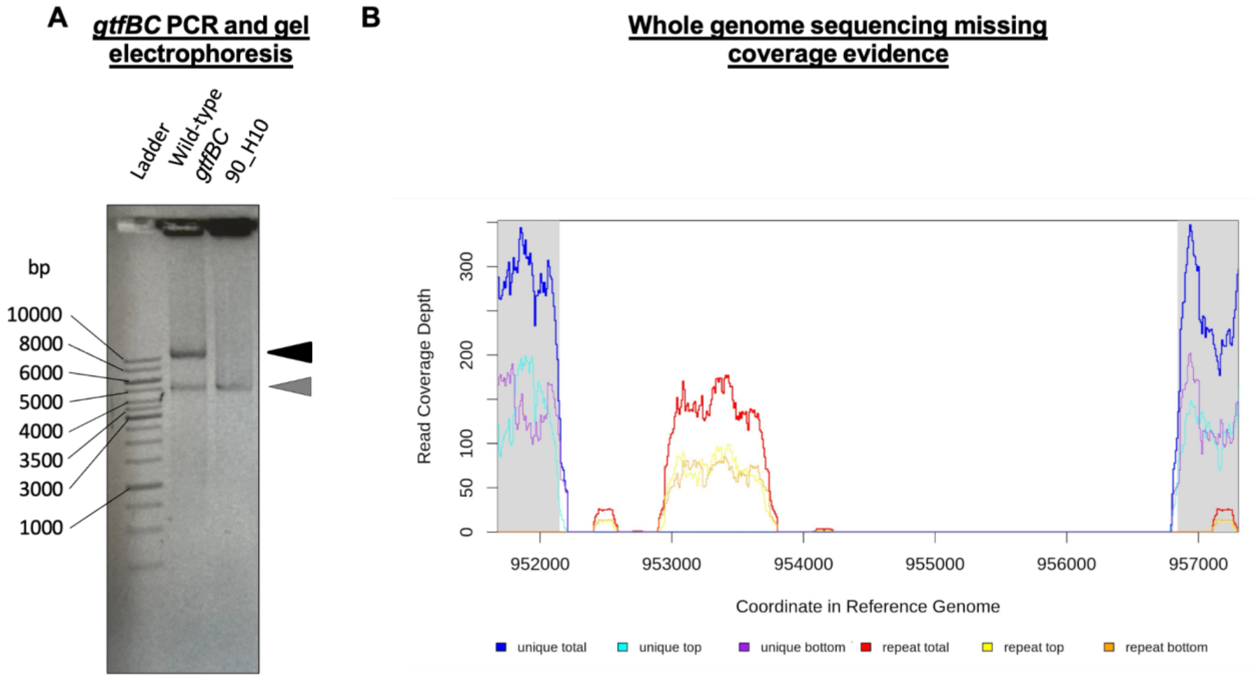

Figure S3 Molecular and genomic evidence of recombination at the *gtfBC* locus for strain 90_H10. (A) To evaluate the genomic integrity of the highly homologous glucosyltransferase genes, PCR was performed using primers targeting the *gtfB* and *gtfC* tandem arrangement. In the wild-type UA159 parent, two characteristic bands are observed at approximately 10 kbp and 4.6 kbp. In the biofilm-defective transposon mutant 90_H10, the absence of the larger 10 kbp product and the presence of a single ~4.6 kbp band confirm a spontaneous large-scale deletion resulting from recombination within the locus. (C) Whole-genome sequencing coverage map of mutant 90_H10. Mapping of sequence reads reveals a definitive lack of coverage across the *gtfBC* genomic coordinates. The absence of read depth in this region provides physical confirmation of the deletion identified by PCR, illustrating that the severe biofilm-deficient phenotype in this strain is a consequence of genomic rearrangement rather than the primary transposon insertion.

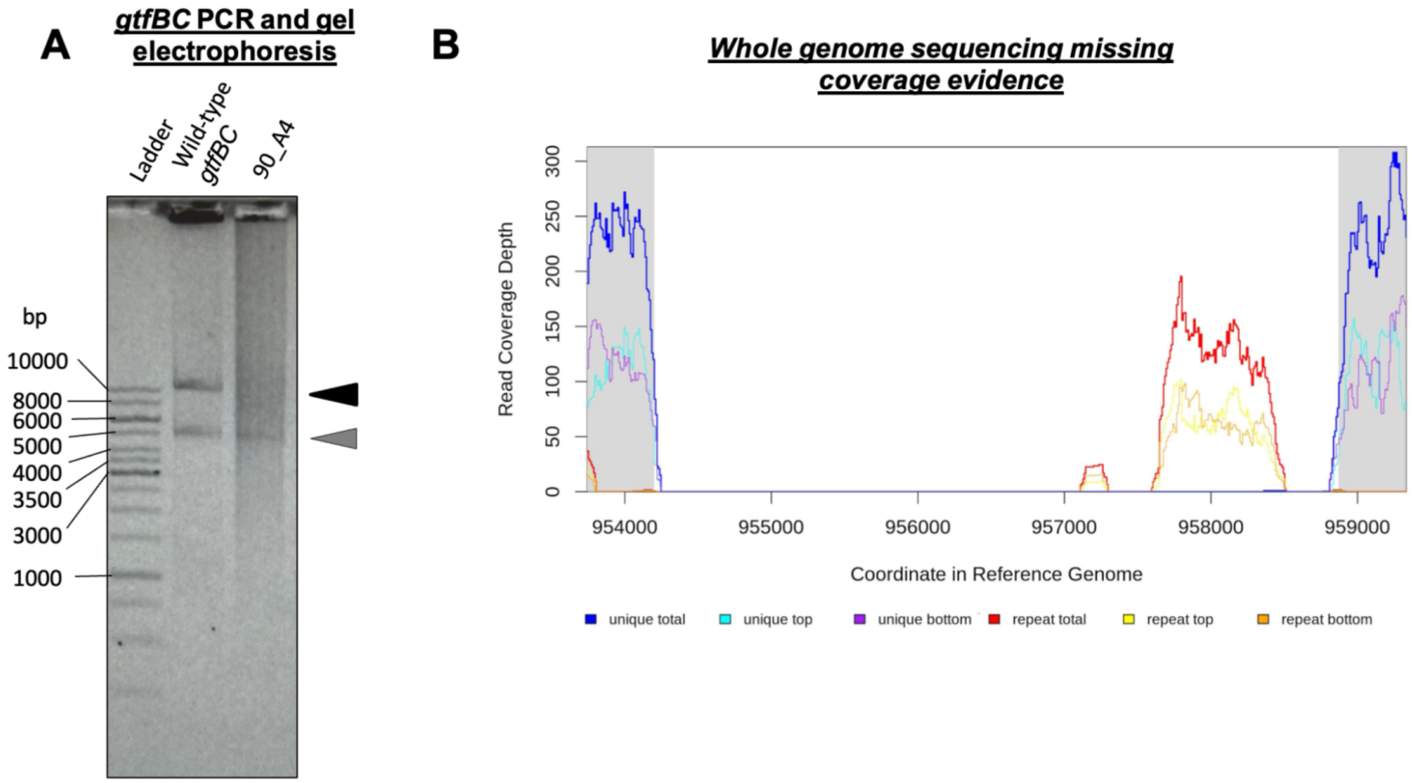

Figure S4 Molecular and genomic evidence of recombination at the *gtfBC* locus for strain 90_A4. (A) To evaluate the genomic integrity of the highly homologous glucosyltransferase genes, PCR was performed using primers targeting the *gtfB* and *gtfC* tandem arrangement. In the wild-type UA159 parent, two characteristic bands are observed at approximately 10 kbp and 4.6 kbp. In the biofilm-defective transposon mutant 90_A4, the absence of the larger 10 kbp product and the presence of a single ~4.6 kbp band confirm a spontaneous large-scale deletion resulting from recombination within the locus. (C) Whole-genome sequencing coverage map of mutant 90_A4. Mapping of sequence reads reveals a definitive lack of coverage across the *gtfBC* genomic coordinates. The absence of read depth in this region provides physical confirmation of the deletion identified by PCR, illustrating that the severe biofilm-deficient phenotype in this strain is a consequence of genomic rearrangement rather than the primary transposon insertion.

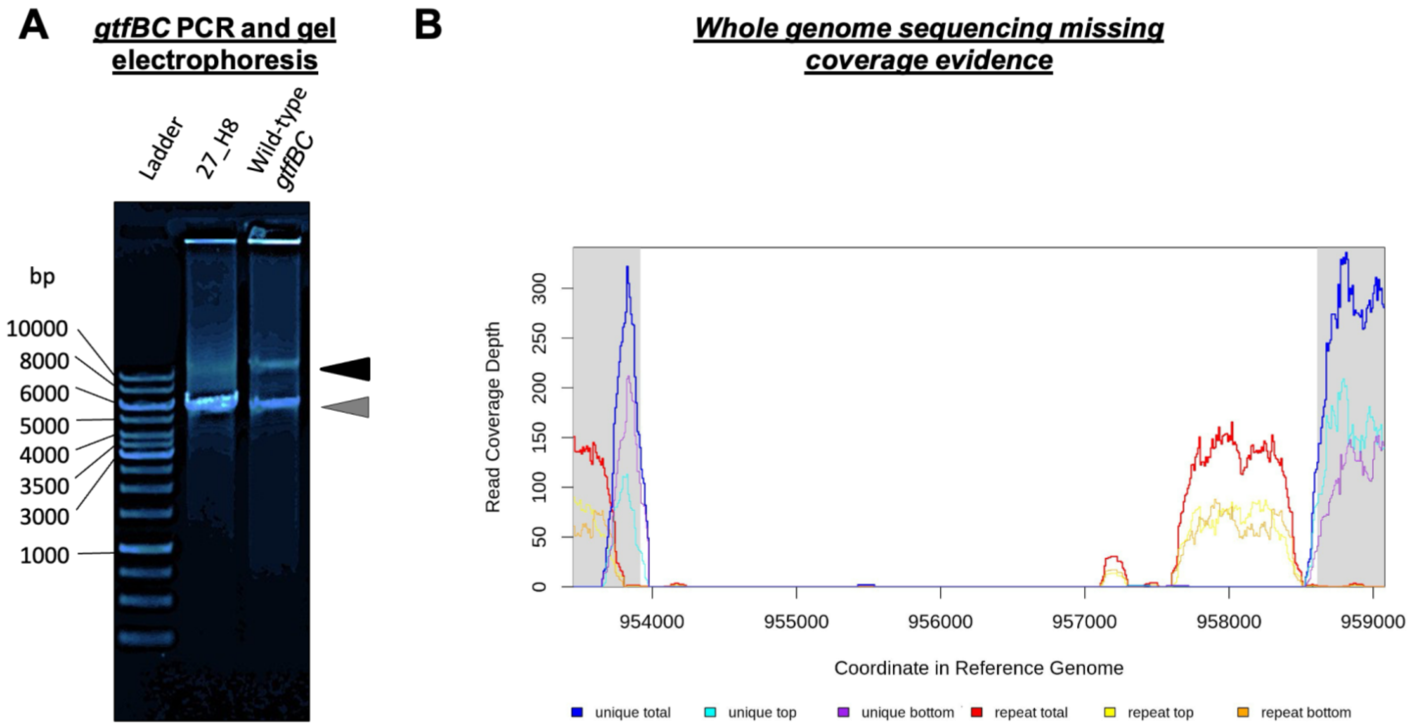

Figure S5 Molecular and genomic evidence of recombination at the *gtfBC* locus for strain 27_H8. (A) To evaluate the genomic integrity of the highly homologous glucosyltransferase genes, PCR was performed using primers targeting the *gtfB* and *gtfC* tandem arrangement. In the wild-type UA159 parent, two characteristic bands are observed at approximately 10 kbp and 4.6 kbp. In the biofilm-defective transposon mutant 27_H8, the absence of the larger 10 kbp product and the presence of a single ~4.6 kbp band confirm a spontaneous large-scale deletion resulting from recombination within the locus. (C) Whole-genome sequencing coverage map of mutant 27_H8. Mapping of sequence reads reveals a definitive lack of coverage across the *gtfBC* genomic coordinates. The absence of read depth in this region provides physical confirmation of the deletion identified by PCR, illustrating that the severe biofilm-deficient phenotype in this strain is a consequence of genomic rearrangement rather than the primary transposon insertion.

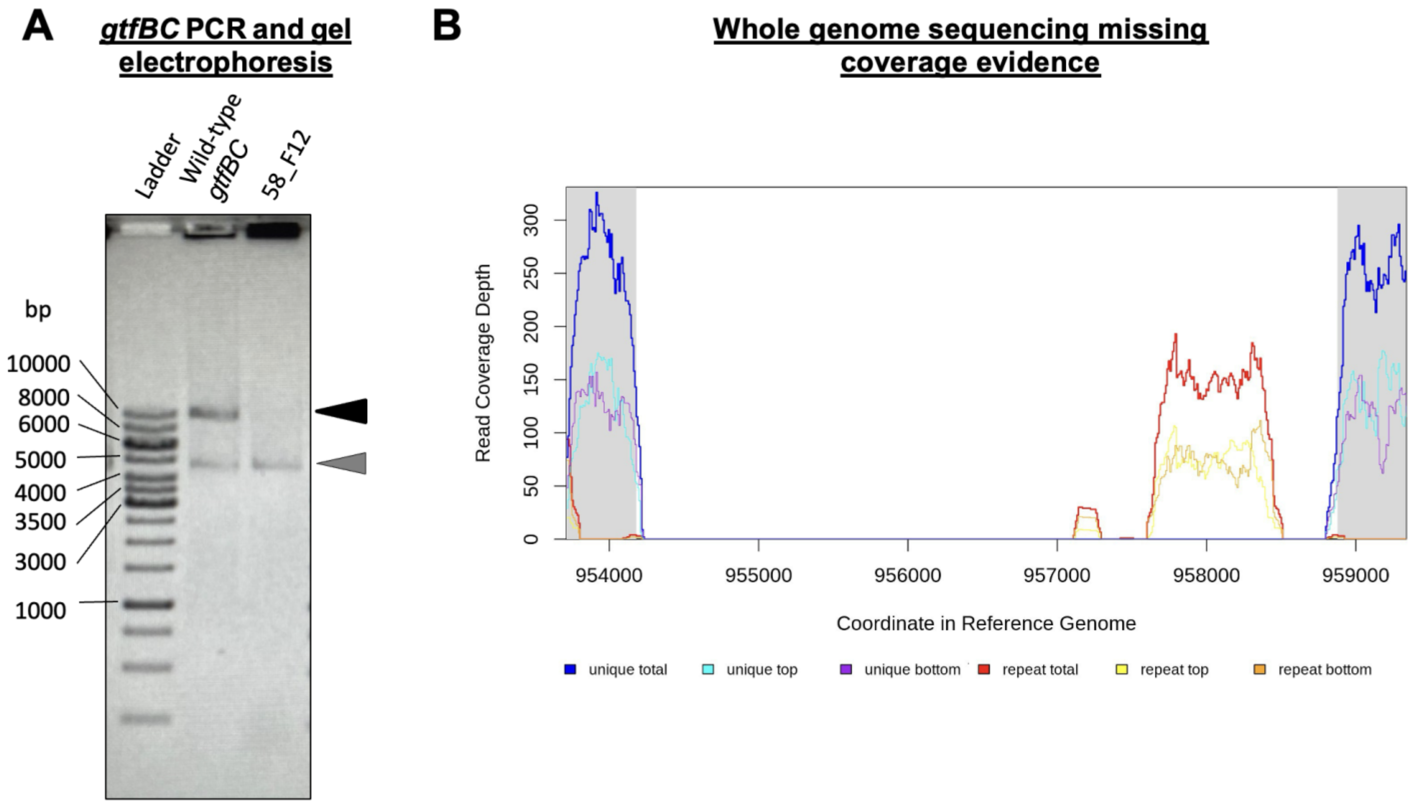

Figure S6 Molecular and genomic evidence of recombination at the *gtfBC* locus for strain 58_F12. (A) To evaluate the genomic integrity of the highly homologous glucosyltransferase genes, PCR was performed using primers targeting the *gtfB* and *gtfC* tandem arrangement. In the wild-type UA159 parent, two characteristic bands are observed at approximately 10 kbp and 4.6 kbp. In the biofilm-defective transposon mutant 58_F12, the absence of the larger 10 kbp product and the presence of a single ~4.6 kbp band confirm a spontaneous large-scale deletion resulting from recombination within the locus. (C) Whole-genome sequencing coverage map of mutant 58_F12. Mapping of sequence reads reveals a definitive lack of coverage across the *gtfBC* genomic coordinates. The absence of read depth in this region provides physical confirmation of the deletion identified by PCR, illustrating that the severe biofilm-deficient phenotype in this strain is a consequence of genomic rearrangement rather than the primary transposon insertion.

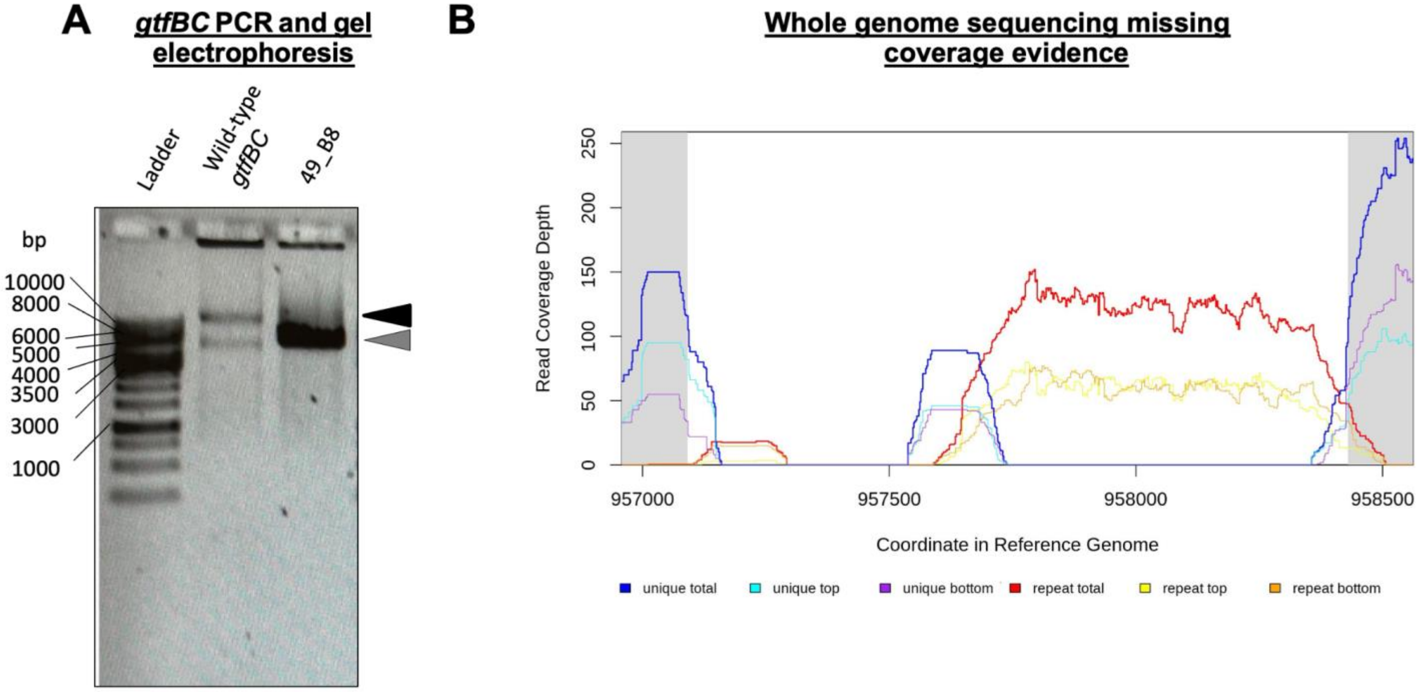

Figure S7 Molecular and genomic evidence of recombination at the *gtfBC* locus for strain 49_B8. (A) To evaluate the genomic integrity of the highly homologous glucosyltransferase genes, PCR was performed using primers targeting the *gtfB* and *gtfC* tandem arrangement. In the wild-type UA159 parent, two characteristic bands are observed at approximately 10 kbp and 4.6 kbp. In the biofilm-defective transposon mutant 49_B8, the absence of the larger 10 kbp product and the presence of a single ~4.6 kbp band confirm a spontaneous large-scale deletion resulting from recombination within the locus. (C) Whole-genome sequencing coverage map of mutant 49_B8. Mapping of sequence reads reveals a definitive lack of coverage across the *gtfBC* genomic coordinates. The absence of read depth in this region provides physical confirmation of the deletion identified by PCR, illustrating that the severe biofilm-deficient phenotype in this strain is a consequence of genomic rearrangement rather than the primary transposon insertion.

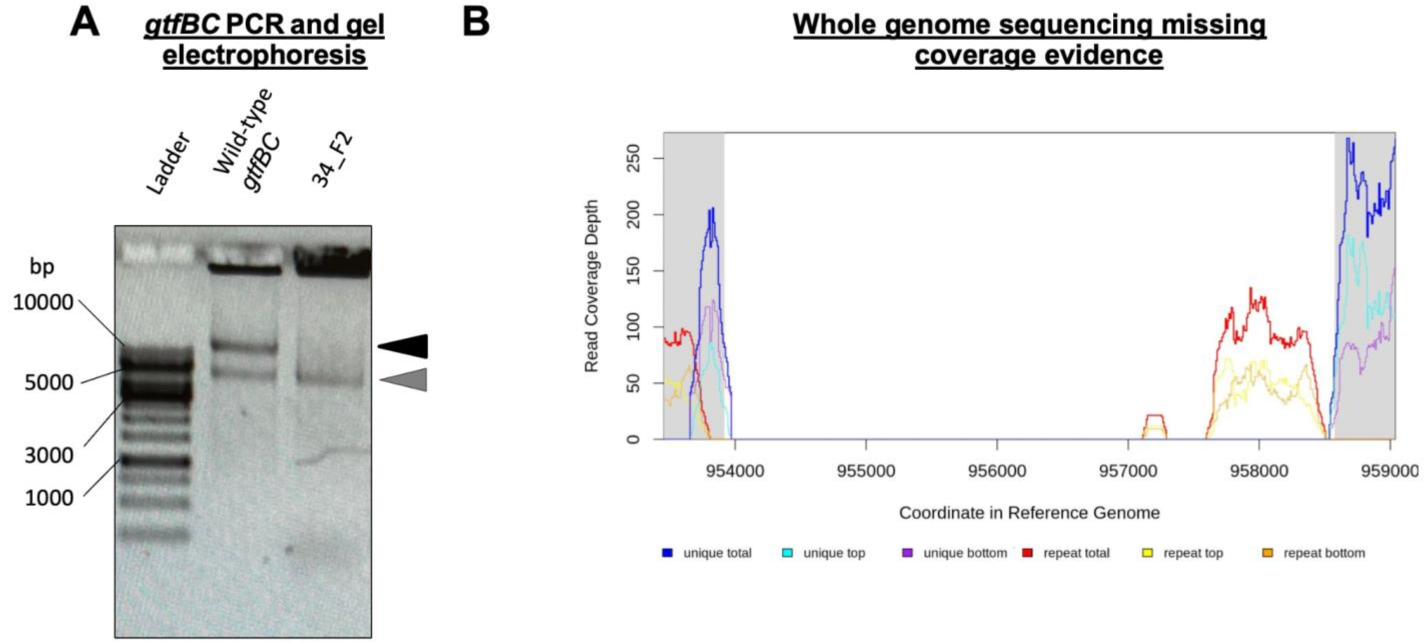

Figure S8 Molecular and genomic evidence of recombination at the *gtfBC* locus for strain 34_F2. (A) To evaluate the genomic integrity of the highly homologous glucosyltransferase genes, PCR was performed using primers targeting the *gtfB* and *gtfC* tandem arrangement. In the wild-type UA159 parent, two characteristic bands are observed at approximately 10 kbp and 4.6 kbp. In the biofilm-defective transposon mutant 34_F2, the absence of the larger 10 kbp product and the presence of a single ~4.6 kbp band confirm a spontaneous large-scale deletion resulting from recombination within the locus. (C) Whole-genome sequencing coverage map of mutant 34_F2. Mapping of sequence reads reveals a definitive lack of coverage across the *gtfBC* genomic coordinates. The absence of read depth in this region provides physical confirmation of the deletion identified by PCR, illustrating that the severe biofilm-deficient phenotype in this strain is a consequence of genomic rearrangement rather than the primary transposon insertion.

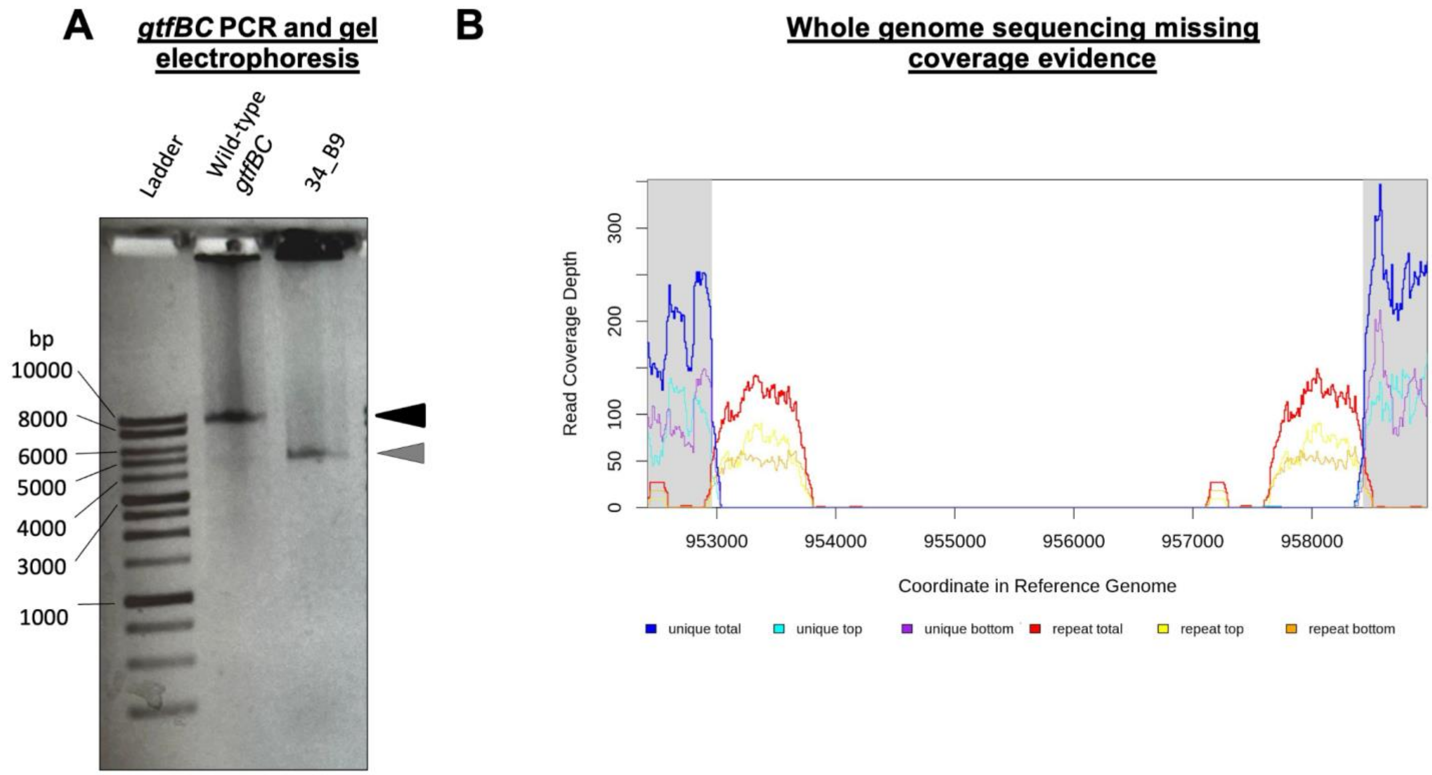

Figure S9 Molecular and genomic evidence of recombination at the *gtfBC* locus for strain 34_B9. (A) To evaluate the genomic integrity of the highly homologous glucosyltransferase genes, PCR was performed using primers targeting the *gtfB* and *gtfC* tandem arrangement. In the wild-type UA159 parent, two characteristic bands are observed at approximately 10 kbp and 4.6 kbp. In the biofilm-defective transposon mutant 34_B9, the absence of the larger 10 kbp product and the presence of a single ~4.6 kbp band confirm a spontaneous large-scale deletion resulting from recombination within the locus. (C) Whole-genome sequencing coverage map of mutant 34_B9. Mapping of sequence reads reveals a definitive lack of coverage across the *gtfBC* genomic coordinates. The absence of read depth in this region provides physical confirmation of the deletion identified by PCR, illustrating that the severe biofilm-deficient phenotype in this strain is a consequence of genomic rearrangement rather than the primary transposon insertion.

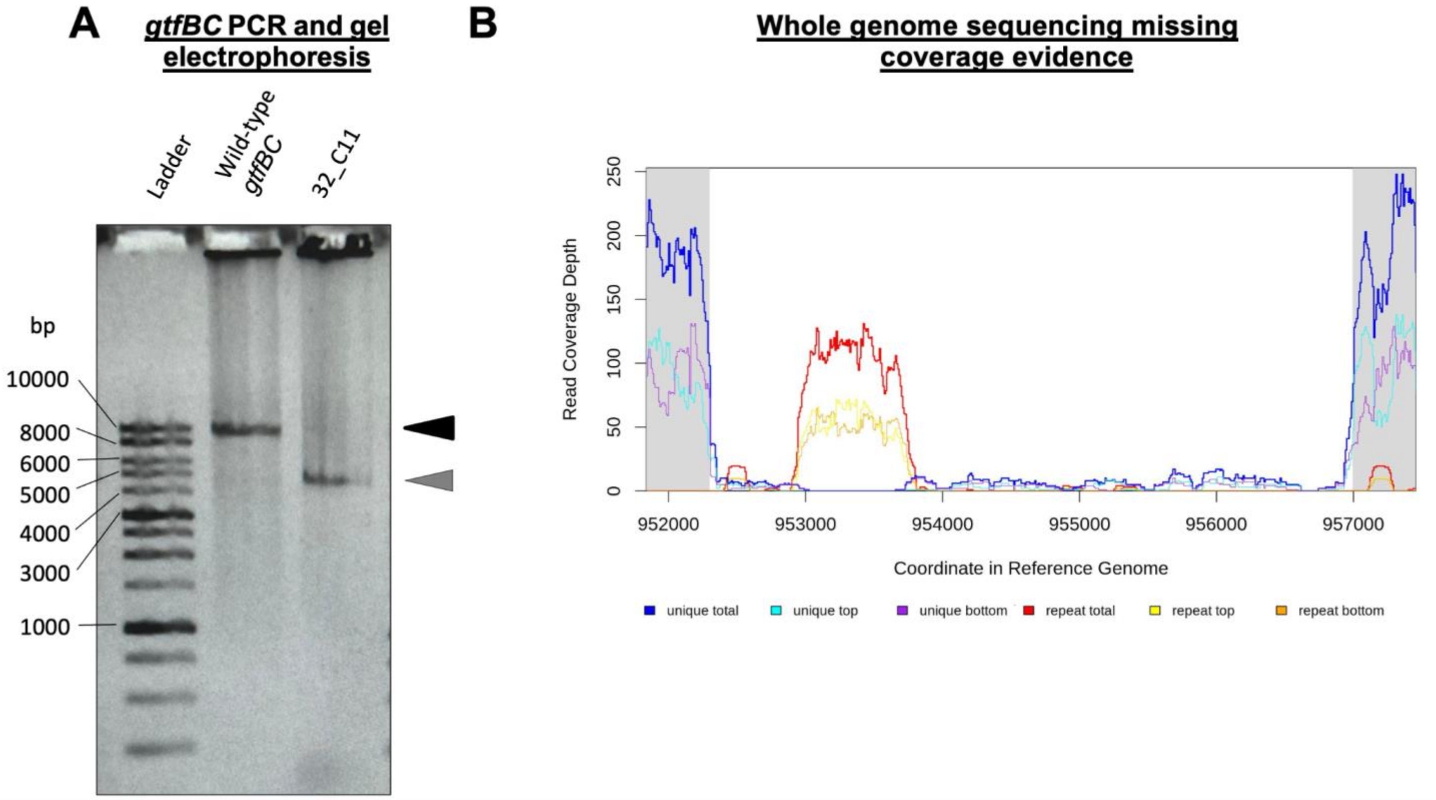

Figure S10 Molecular and genomic evidence of recombination at the *gtfBC* locus for strain 32_C11. (A) To evaluate the genomic integrity of the highly homologous glucosyltransferase genes, PCR was performed using primers targeting the *gtfB* and *gtfC* tandem arrangement. In the wild-type UA159 parent, two characteristic bands are observed at approximately 10 kbp and 4.6 kbp. In the biofilm-defective transposon mutant 32_C11, the absence of the larger 10 kbp product and the presence of a single ~4.6 kbp band confirm a spontaneous large-scale deletion resulting from recombination within the locus. (C) Whole-genome sequencing coverage map of mutant 32_C11. Mapping of sequence reads reveals a definitive lack of coverage across the *gtfBC* genomic coordinates. The absence of read depth in this region provides physical confirmation of the deletion identified by PCR, illustrating that the severe biofilm-deficient phenotype in this strain is a consequence of genomic rearrangement rather than the primary transposon insertion.

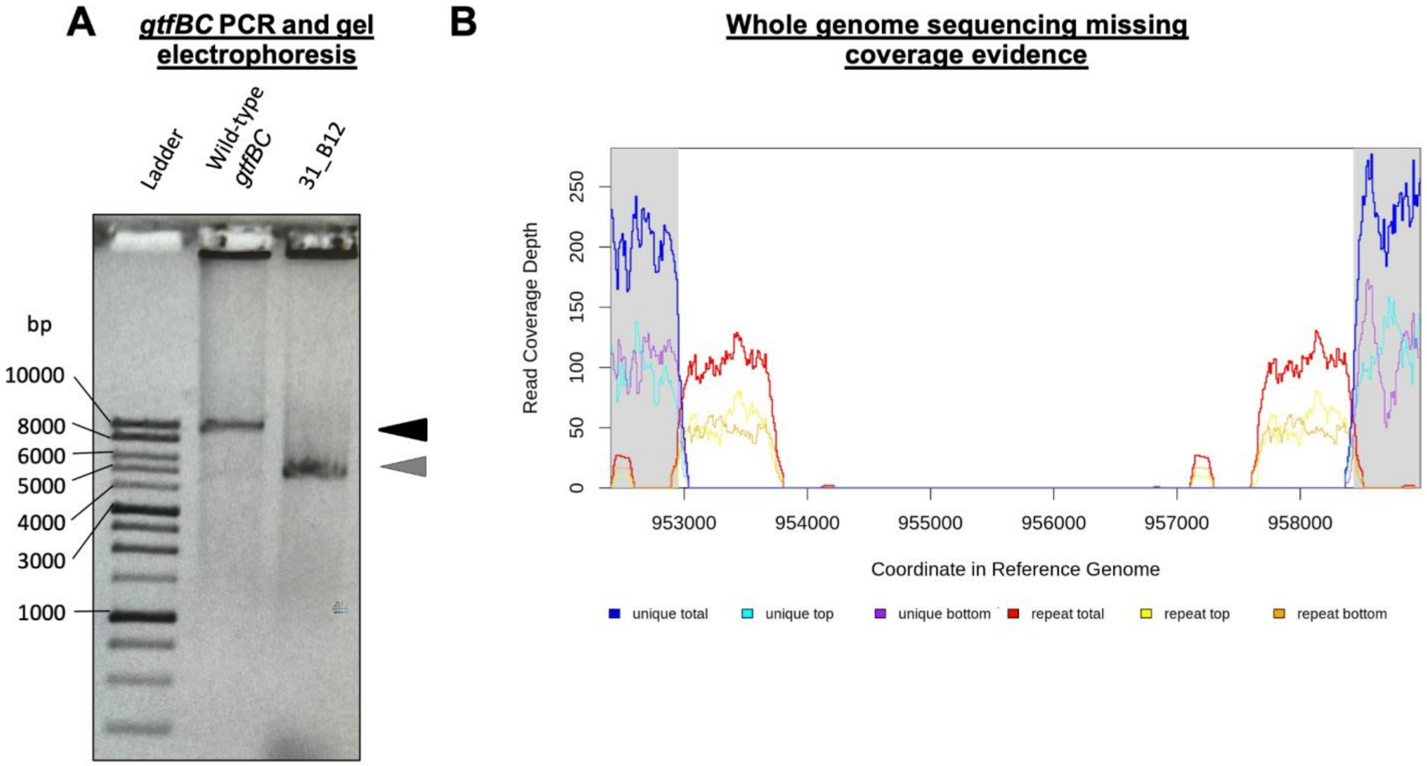

Figure S11 Molecular and genomic evidence of recombination at the *gtfBC* locus for strain 31_B12. (A) To evaluate the genomic integrity of the highly homologous glucosyltransferase genes, PCR was performed using primers targeting the *gtfB* and *gtfC* tandem arrangement. In the wild-type UA159 parent, two characteristic bands are observed at approximately 10 kbp and 4.6 kbp. In the biofilm-defective transposon mutant 31_B12, the absence of the larger 10 kbp product and the presence of a single ~4.6 kbp band confirm a spontaneous large-scale deletion resulting from recombination within the locus. (C) Whole-genome sequencing coverage map of mutant 31_B12. Mapping of sequence reads reveals a definitive lack of coverage across the *gtfBC* genomic coordinates. The absence of read depth in this region provides physical confirmation of the deletion identified by PCR, illustrating that the severe biofilm-deficient phenotype in this strain is a consequence of genomic rearrangement rather than the primary transposon insertion.

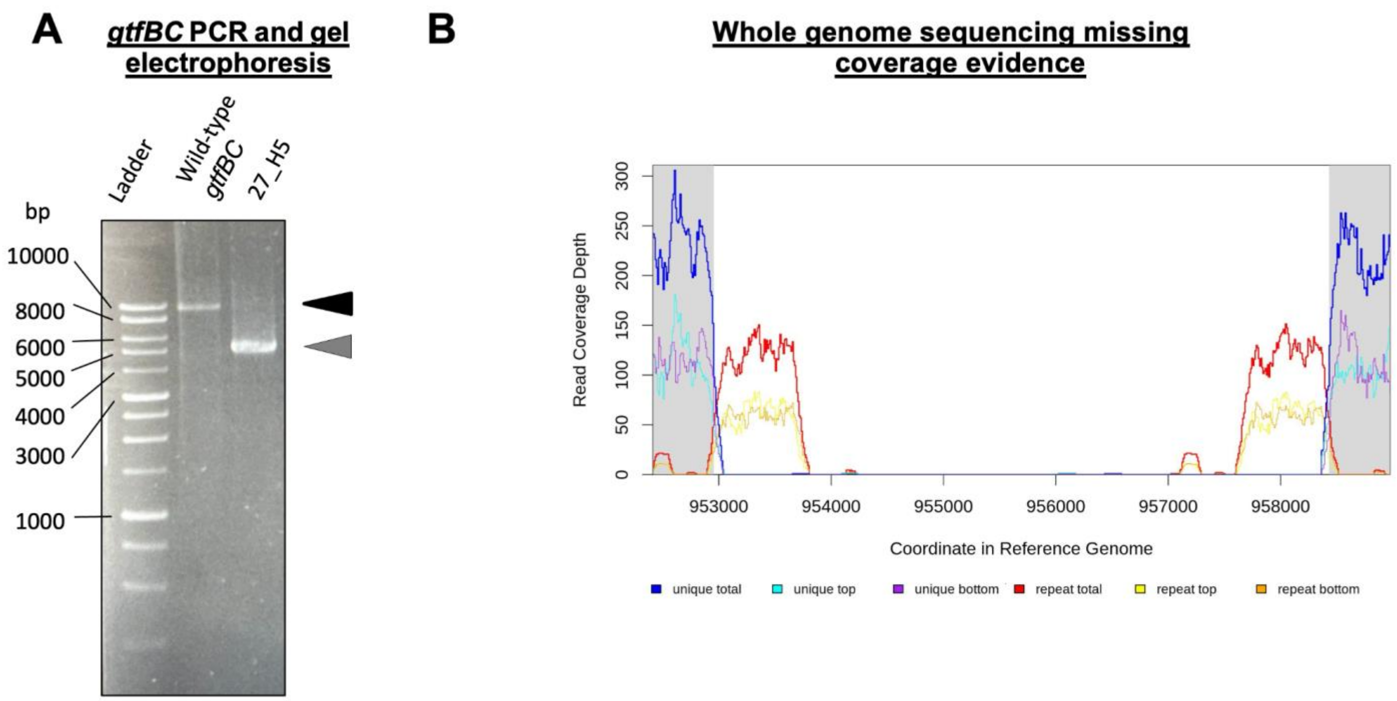

Figure S12 Molecular and genomic evidence of recombination at the *gtfBC* locus for strain 27_H5. (A) To evaluate the genomic integrity of the highly homologous glucosyltransferase genes, PCR was performed using primers targeting the *gtfB* and *gtfC* tandem arrangement. In the wild-type UA159 parent, two characteristic bands are observed at approximately 10 kbp and 4.6 kbp. In the biofilm-defective transposon mutant 27_H5, the absence of the larger 10 kbp product and the presence of a single ~4.6 kbp band confirm a spontaneous large-scale deletion resulting from recombination within the locus. (C) Whole-genome sequencing coverage map of mutant 27_H5. Mapping of sequence reads reveals a definitive lack of coverage across the *gtfBC* genomic coordinates. The absence of read depth in this region provides physical confirmation of the deletion identified by PCR, illustrating that the severe biofilm-deficient phenotype in this strain is a consequence of genomic rearrangement rather than the primary transposon insertion.

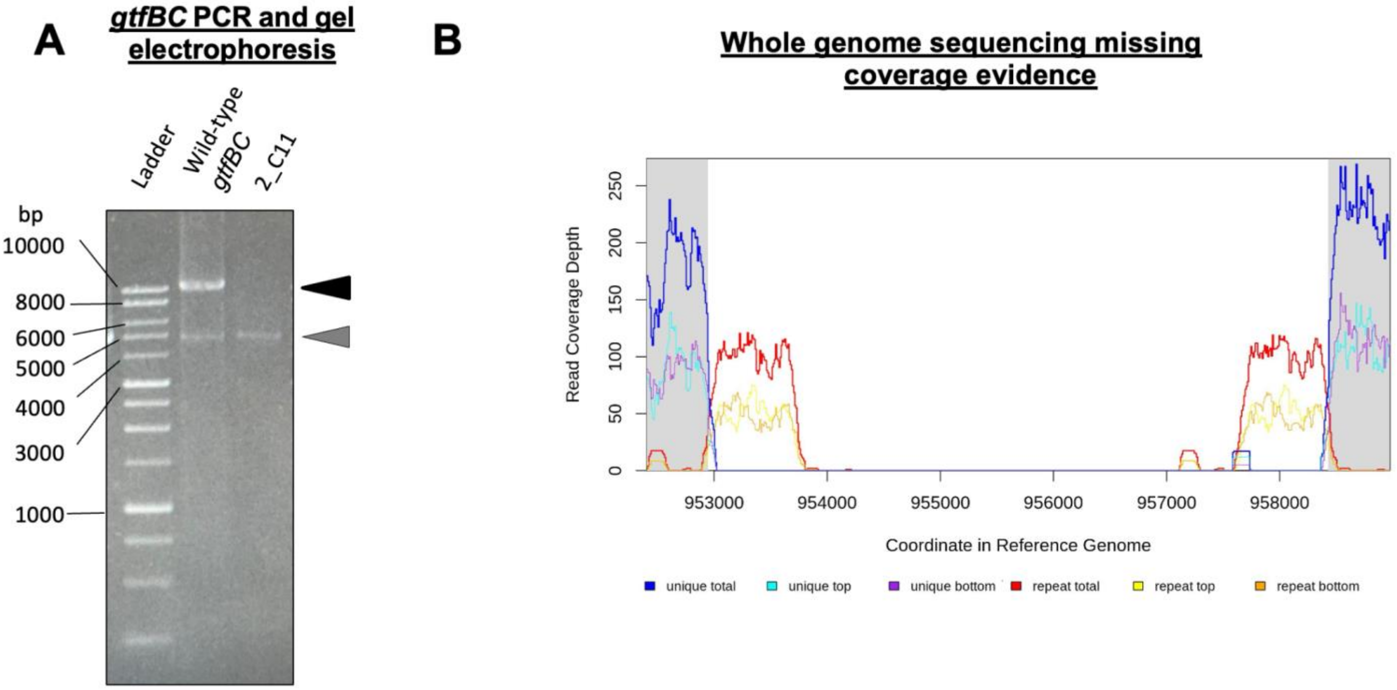

Figure S13 Molecular and genomic evidence of recombination at the *gtfBC* locus for strain 2_C11. (A) To evaluate the genomic integrity of the highly homologous glucosyltransferase genes, PCR was performed using primers targeting the *gtfB* and *gtfC* tandem arrangement. In the wild-type UA159 parent, two characteristic bands are observed at approximately 10 kbp and 4.6 kbp. In the biofilm-defective transposon mutant 2_C11, the absence of the larger 10 kbp product and the presence of a single ~4.6 kbp band confirm a spontaneous large-scale deletion resulting from recombination within the locus. (C) Whole-genome sequencing coverage map of mutant 2_C11. Mapping of sequence reads reveals a definitive lack of coverage across the *gtfBC* genomic coordinates. The absence of read depth in this region provides physical confirmation of the deletion identified by PCR, illustrating that the severe biofilm-deficient phenotype in this strain is a consequence of genomic rearrangement rather than the primary transposon insertion.

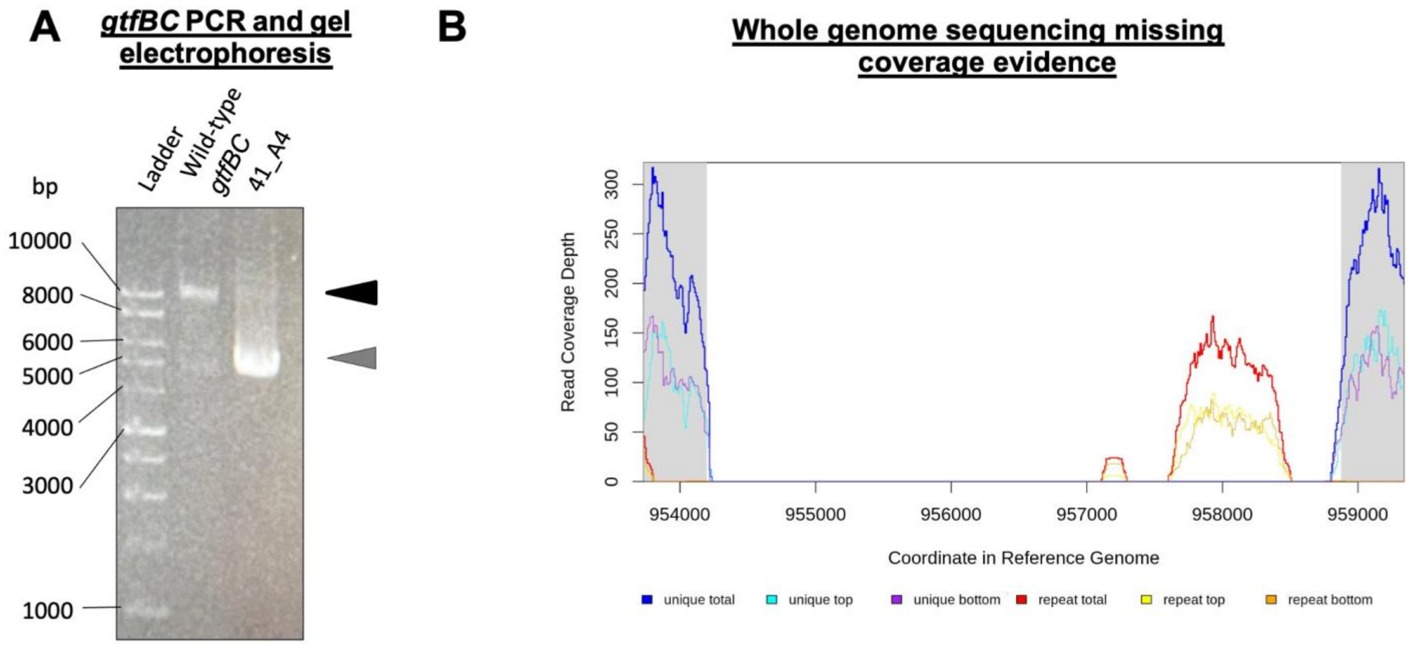

Figure S14 Molecular and genomic evidence of recombination at the *gtfBC* locus for strain 41_A4. (A) To evaluate the genomic integrity of the highly homologous glucosyltransferase genes, PCR was performed using primers targeting the *gtfB* and *gtfC* tandem arrangement. In the wild-type UA159 parent, two characteristic bands are observed at approximately 10 kbp and 4.6 kbp. In the biofilm-defective transposon mutant 41_A4, the absence of the larger 10 kbp product and the presence of a single ~4.6 kbp band confirm a spontaneous large-scale deletion resulting from recombination within the locus. (C) Whole-genome sequencing coverage map of mutant 41_A4. Mapping of sequence reads reveals a definitive lack of coverage across the *gtfBC* genomic coordinates. The absence of read depth in this region provides physical confirmation of the deletion identified by PCR, illustrating that the severe biofilm-deficient phenotype in this strain is a consequence of genomic rearrangement rather than the primary transposon insertion.

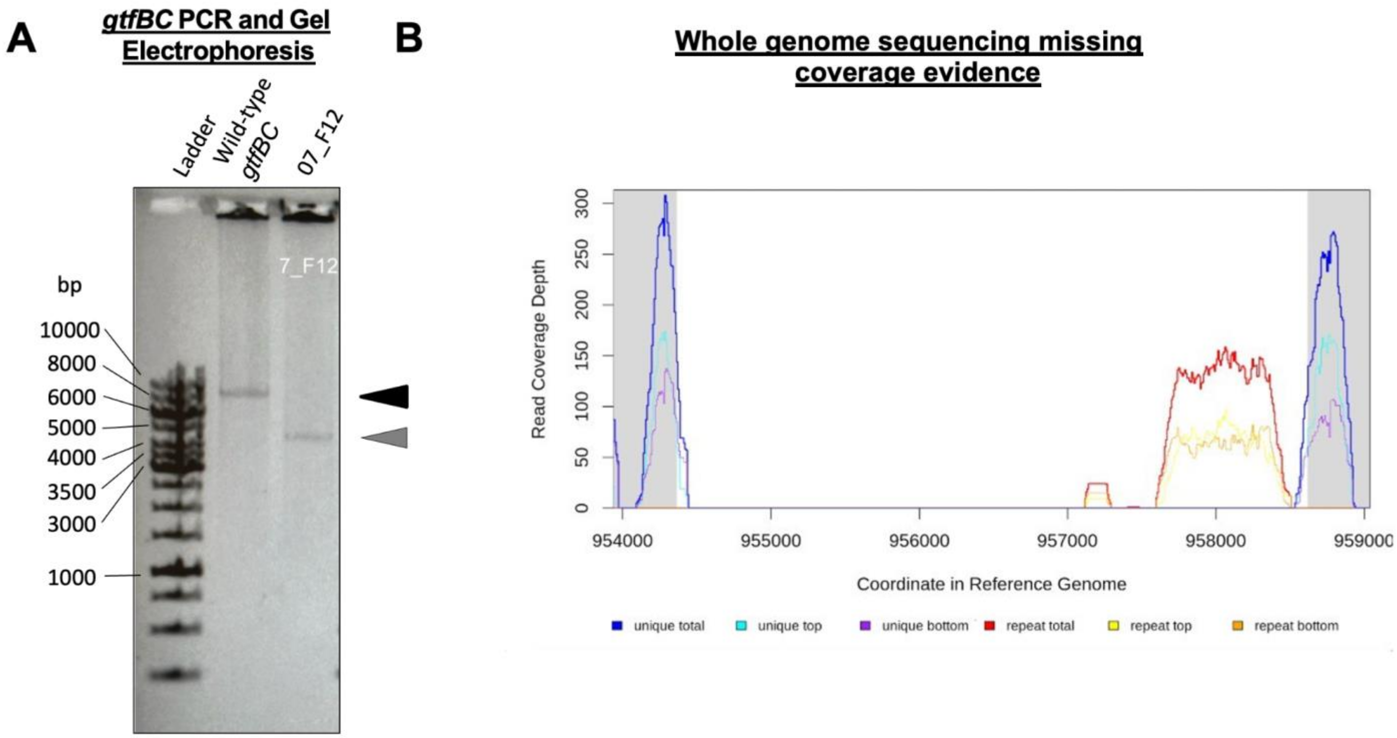

Figure S15 Molecular and genomic evidence of recombination at the *gtfBC* locus for strain 07_F12. (A) To evaluate the genomic integrity of the highly homologous glucosyltransferase genes, PCR was performed using primers targeting the *gtfB* and *gtfC* tandem arrangement. In the wild-type UA159 parent, two characteristic bands are observed at approximately 10 kbp and 4.6 kbp. In the biofilm-defective transposon mutant 07_F12, the absence of the larger 10 kbp product and the presence of a single ~4.6 kbp band confirm a spontaneous large-scale deletion resulting from recombination within the locus. (C) Whole-genome sequencing coverage map of mutant 07_F12. Mapping of sequence reads reveals a definitive lack of coverage across the *gtfBC* genomic coordinates. The absence of read depth in this region provides physical confirmation of the deletion identified by PCR, illustrating that the severe biofilm-deficient phenotype in this strain is a consequence of genomic rearrangement rather than the primary transposon insertion.

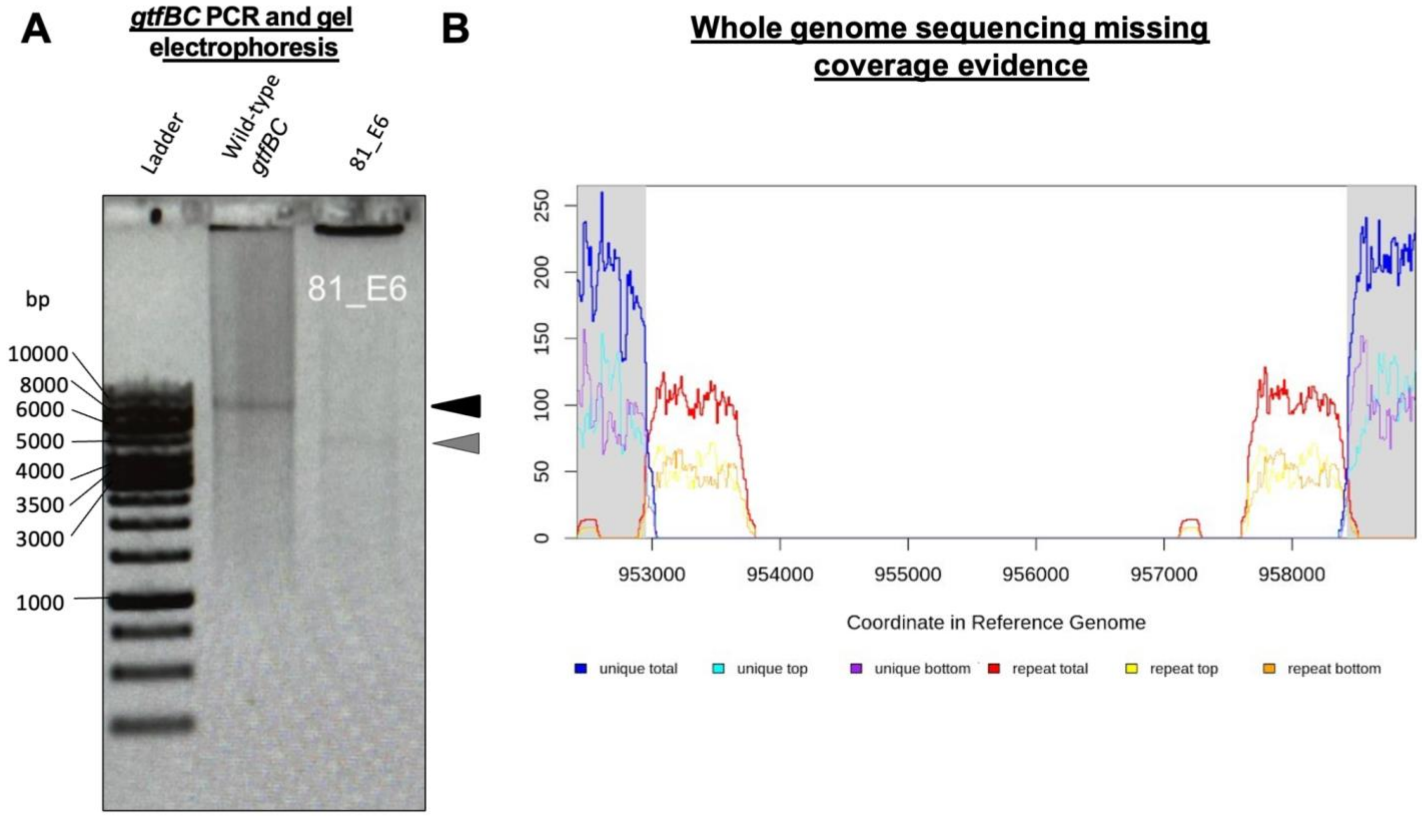

Figure S16 Molecular and genomic evidence of recombination at the *gtfBC* locus for strain 81_E6. (A) To evaluate the genomic integrity of the highly homologous glucosyltransferase genes, PCR was performed using primers targeting the *gtfB* and *gtfC* tandem arrangement. In the wild-type UA159 parent, two characteristic bands are observed at approximately 10 kbp and 4.6 kbp. In the biofilm-defective transposon mutant 81_E6, the absence of the larger 10 kbp product and the presence of a single ~4.6 kbp band confirm a spontaneous large-scale deletion resulting from recombination within the locus. (C) Whole-genome sequencing coverage map of mutant 81_E6. Mapping of sequence reads reveals a definitive lack of coverage across the *gtfBC* genomic coordinates. The absence of read depth in this region provides physical confirmation of the deletion identified by PCR, illustrating that the severe biofilm-deficient phenotype in this strain is a consequence of genomic rearrangement rather than the primary transposon insertion.

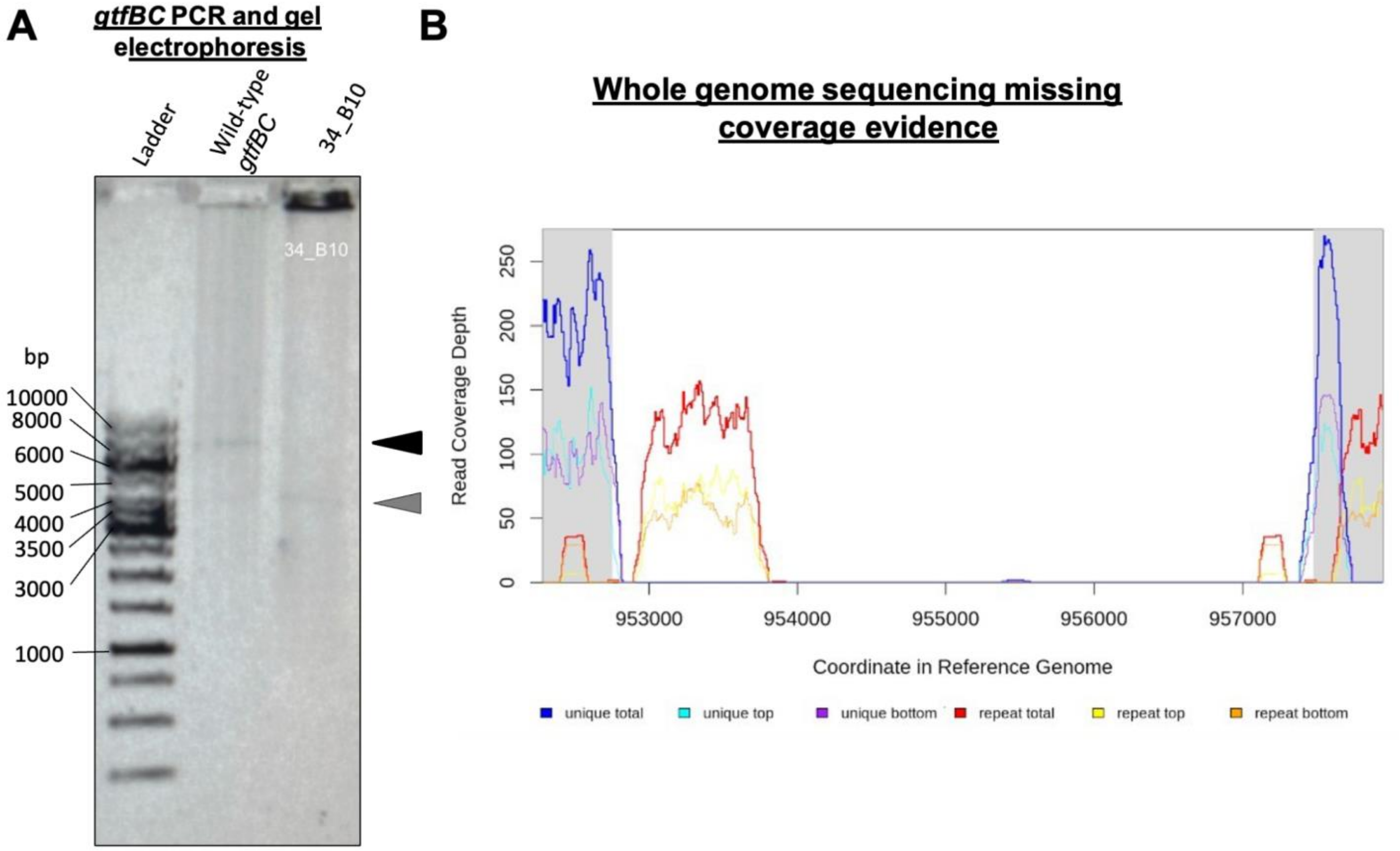

Figure S17 Molecular and genomic evidence of recombination at the *gtfBC* locus for strain 34_B10. (A) To evaluate the genomic integrity of the highly homologous glucosyltransferase genes, PCR was performed using primers targeting the *gtfB* and *gtfC* tandem arrangement. In the wild-type UA159 parent, two characteristic bands are observed at approximately 10 kbp and 4.6 kbp. In the biofilm-defective transposon mutant 34_B10, the absence of the larger 10 kbp product and the presence of a single ~4.6 kbp band confirm a spontaneous large-scale deletion resulting from recombination within the locus. (C) Whole-genome sequencing coverage map of mutant 34_B10. Mapping of sequence reads reveals a definitive lack of coverage across the *gtfBC* genomic coordinates. The absence of read depth in this region provides physical confirmation of the deletion identified by PCR, illustrating that the severe biofilm-deficient phenotype in this strain is a consequence of genomic rearrangement rather than the primary transposon insertion.

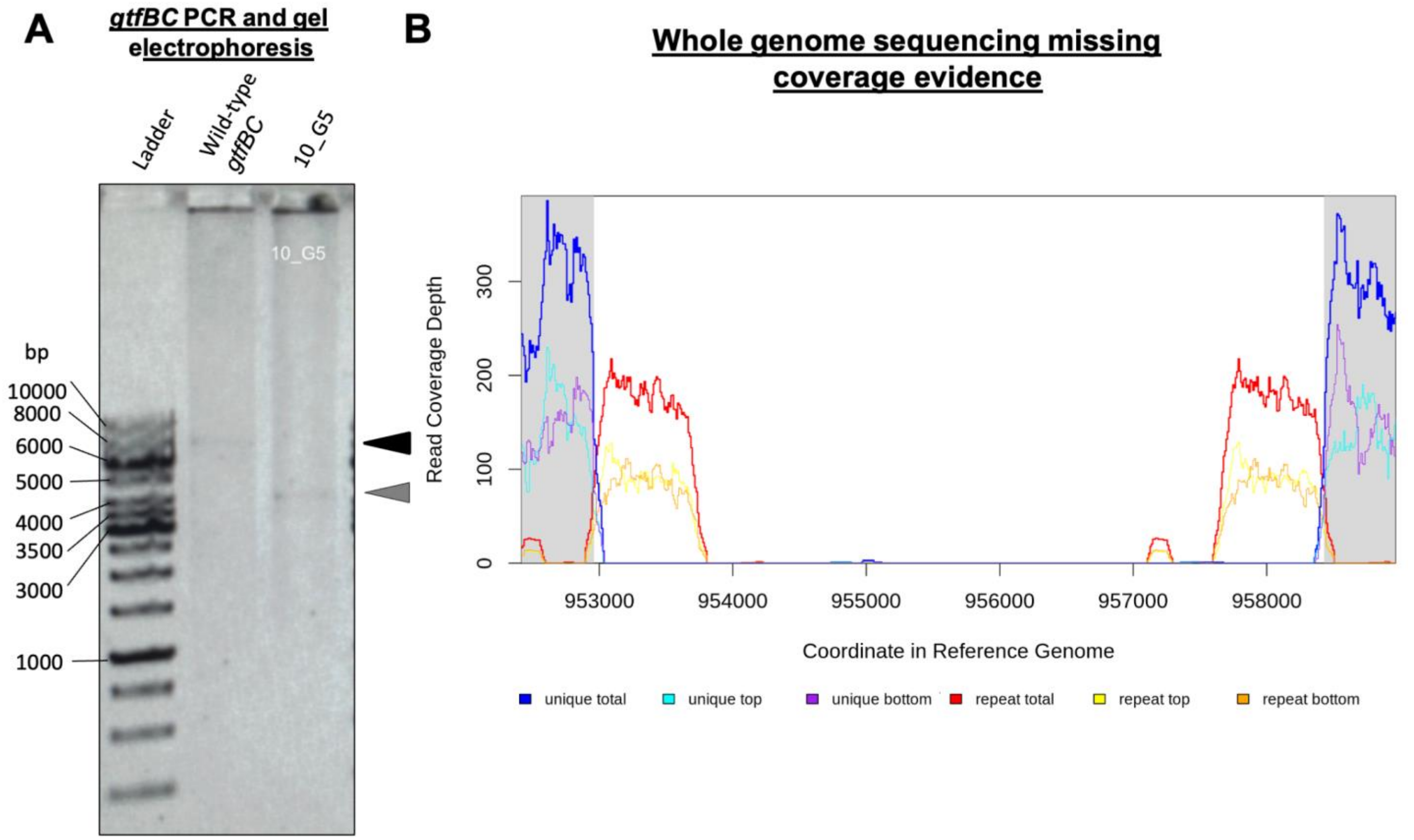

Figure S18 Molecular and genomic evidence of recombination at the *gtfBC* locus for strain 10_G5. (A) To evaluate the genomic integrity of the highly homologous glucosyltransferase genes, PCR was performed using primers targeting the *gtfB* and *gtfC* tandem arrangement. In the wild-type UA159 parent, two characteristic bands are observed at approximately 10 kbp and 4.6 kbp. In the biofilm-defective transposon mutant 10_G5, the absence of the larger 10 kbp product and the presence of a single ~4.6 kbp band confirm a spontaneous large-scale deletion resulting from recombination within the locus. (C) Whole-genome sequencing coverage map of mutant 10_G5. Mapping of sequence reads reveals a definitive lack of coverage across the *gtfBC* genomic coordinates. The absence of read depth in this region provides physical confirmation of the deletion identified by PCR, illustrating that the severe biofilm-deficient phenotype in this strain is a consequence of genomic rearrangement rather than the primary transposon insertion.

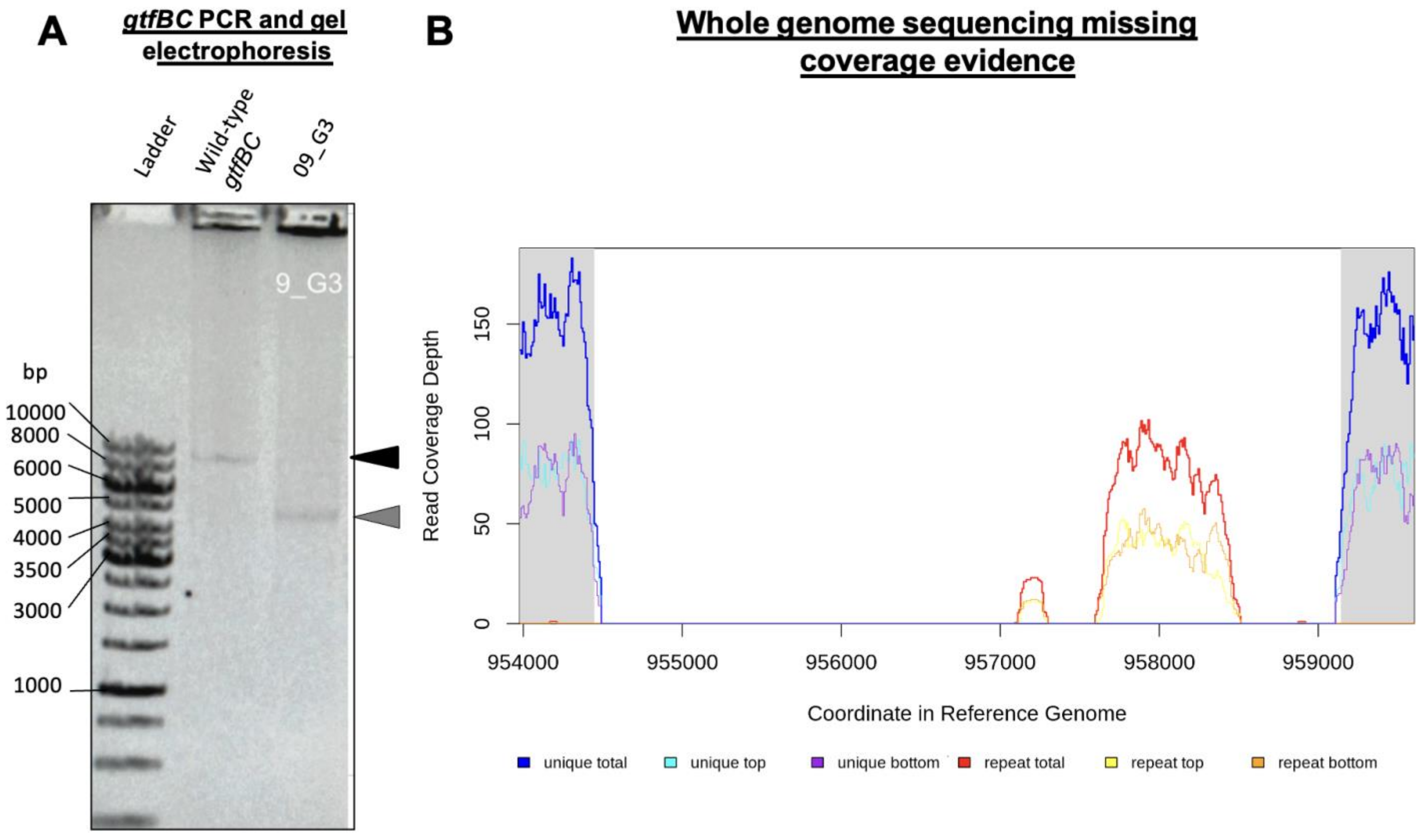

Figure S19 Molecular and genomic evidence of recombination at the *gtfBC* locus for strain 09_G3. (A) To evaluate the genomic integrity of the highly homologous glucosyltransferase genes, PCR was performed using primers targeting the *gtfB* and *gtfC* tandem arrangement. In the wild-type UA159 parent, two characteristic bands are observed at approximately 10 kbp and 4.6 kbp. In the biofilm-defective transposon mutant 09_G3, the absence of the larger 10 kbp product and the presence of a single ~4.6 kbp band confirm a spontaneous large-scale deletion resulting from recombination within the locus. (C) Whole-genome sequencing coverage map of mutant 09_G3. Mapping of sequence reads reveals a definitive lack of coverage across the *gtfBC* genomic coordinates. The absence of read depth in this region provides physical confirmation of the deletion identified by PCR, illustrating that the severe biofilm-deficient phenotype in this strain is a consequence of genomic rearrangement rather than the primary transposon insertion.

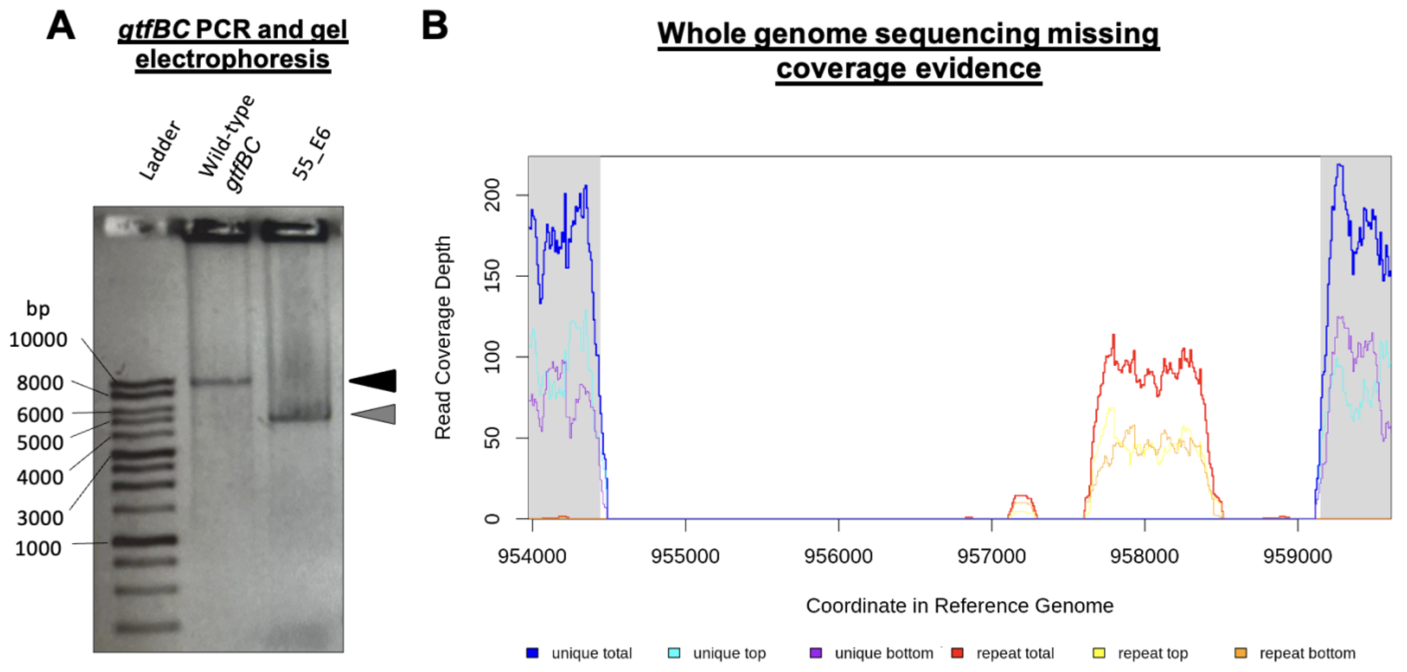

Figure S20 Molecular and genomic evidence of recombination at the *gtfBC* locus for strain 55_E6. (A) To evaluate the genomic integrity of the highly homologous glucosyltransferase genes, PCR was performed using primers targeting the *gtfB* and *gtfC* tandem arrangement. In the wild-type UA159 parent, two characteristic bands are observed at approximately 10 kbp and 4.6 kbp. In the biofilm-defective transposon mutant 55_E6, the absence of the larger 10 kbp product and the presence of a single ~4.6 kbp band confirm a spontaneous large-scale deletion resulting from recombination within the locus. (C) Whole-genome sequencing coverage map of mutant 55_E6. Mapping of sequence reads reveals a definitive lack of coverage across the *gtfBC* genomic coordinates. The absence of read depth in this region provides physical confirmation of the deletion identified by PCR, illustrating that the severe biofilm-deficient phenotype in this strain is a consequence of genomic rearrangement rather than the primary transposon insertion.

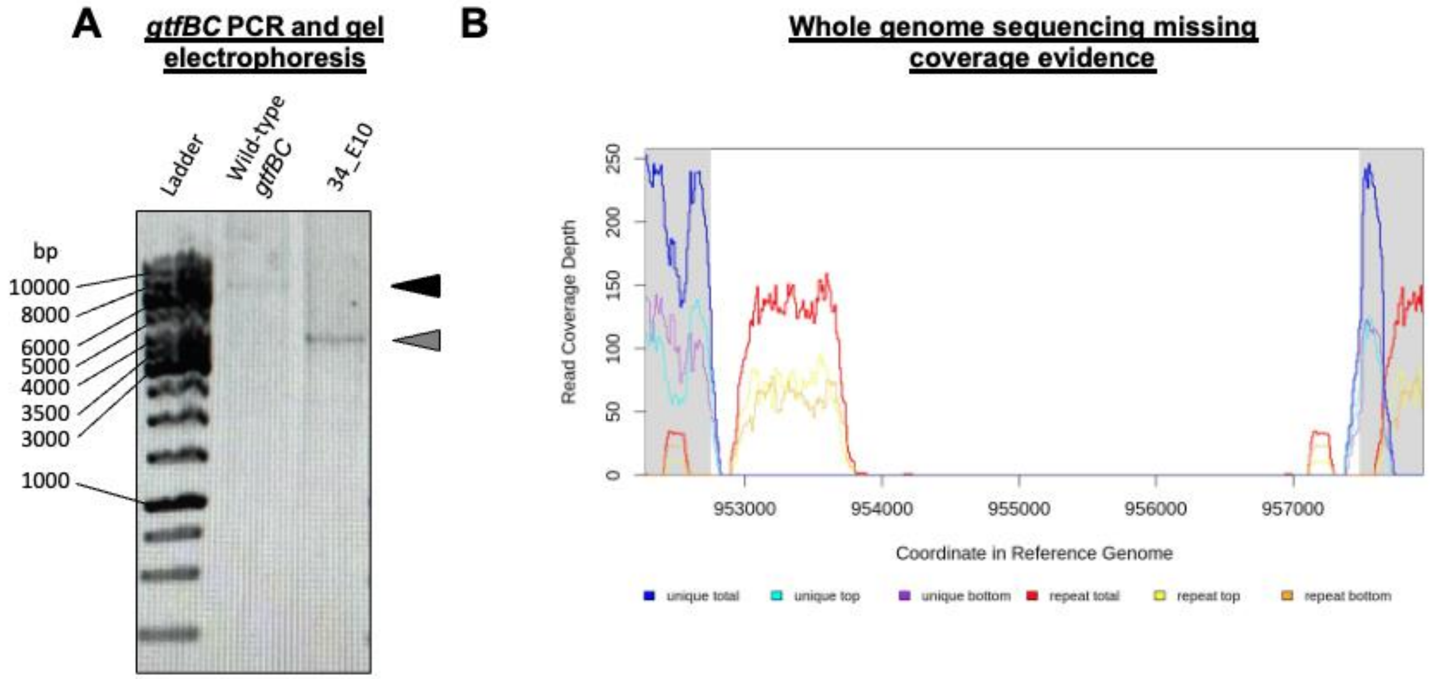

Figure S21 Molecular and genomic evidence of recombination at the *gtfBC* locus for strain 34_E10. (A) To evaluate the genomic integrity of the highly homologous glucosyltransferase genes, PCR was performed using primers targeting the *gtfB* and *gtfC* tandem arrangement. In the wild-type UA159 parent, two characteristic bands are observed at approximately 10 kbp and 4.6 kbp. In the biofilm-defective transposon mutant 34_E10, the absence of the larger 10 kbp product and the presence of a single ~4.6 kbp band confirm a spontaneous large-scale deletion resulting from recombination within the locus. (C) Whole-genome sequencing coverage map of mutant 34_E10. Mapping of sequence reads reveals a definitive lack of coverage across the *gtfBC* genomic coordinates. The absence of read depth in this region provides physical confirmation of the deletion identified by PCR, illustrating that the severe biofilm-deficient phenotype in this strain is a consequence of genomic rearrangement rather than the primary transposon insertion.

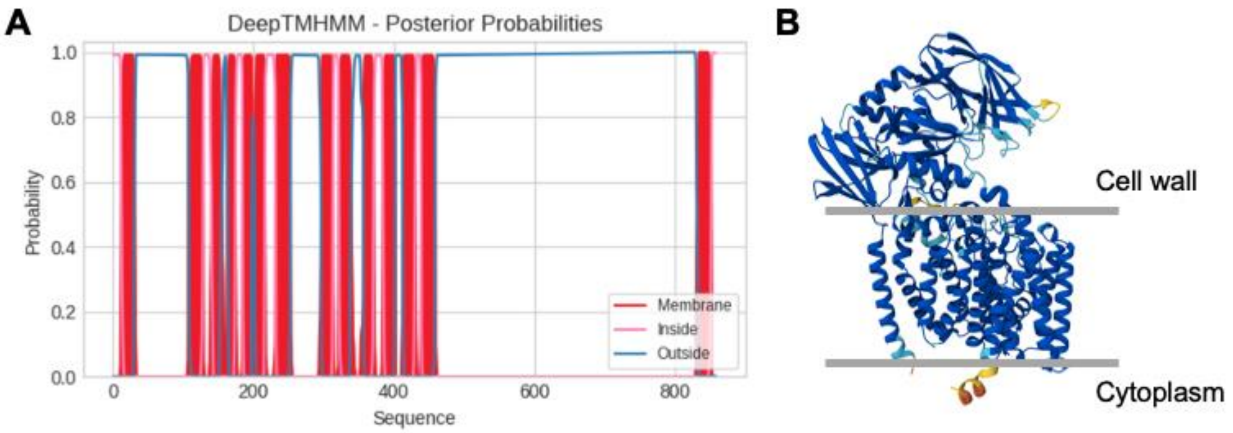

Figure S22 Predicted topology and structural model of SMU_2160. (A) Membrane topology prediction using DeepTMHMM. The posterior probability plot for the 857-amino acid sequence of SMU_2160 predicts thirteen transmembrane (TM) helices (shown in red). The analysis identifies a large extracellular domain (shown in blue) of approximately 375 amino acids. (B) An AlphaFold tertiary structure model illustrates the thirteen-TM helical bundle embedded within the membrane, spanning the cell membrane. The large extracellular domain is shown extending toward the cell wall, where it is hypothesized to facilitate the capture and transfer of carbohydrate moieties for surface protein or cell wall glycosylation.

Table S2 Whole genome sequencing of transposon insertions mutants with sucrose-mediated biofilm defects

Strain	Location of Tn insertion	Gene name	Gene description	Predicted mutations†	Missing coverage evidence*	Link to sugar utilization or biofilm formation	Z-score
09_G2	SMU_47		HNH endonuclease signature motif containing protein	Intergenic (+353/-30), SMU_60 → / → *comR* G89G (GGG→GGA) *scnE*	SMU_436c; SMU_565c–SMU_566c		-5.83
57_D1	Intergenic between SMU_1924 - SMU_1925	*gcrR* - SMU_1925c	Orphan response regulator; DUF177 domain-containing protein		SMU_436c; *ΔTnSmu1*	PMID: 16428403	-5.52
82_G1	SMU_1924-SMU_1925c	*gcrR* - SMU_1925c	Orphan response regulator; DUF177 domain-containing protein	T60M (ACG→ATG) *comR*		PMID: 16428403	-5.20
55_B1	Intergenic between SMU_429c - SMU_431		Inner membrane protein; ATP-binding protein	V72I (GTT→ATT) *cpsY*	SMU_436c		-4.59
58_F8	SMU_1660c	*yabA*	DNA replication initiation control protein	Intergenic (+353/-30) SMU_60 → / → *comR*			-4.47
63_F12	Intergenic between SMU_1924 - SMU_1925	*gcrR* - SMU_1925c	Orphan response regulator; DUF177 domain-containing protein	Intergenic (+353/-30) SMU_60 → / → SMU_61; Y202Y (TAT→TAC) SMU_1370c; S201P (TCA→CCA) SMU_1370c ; E200K (GAG→AAG) SMU_1370c	SMU_1370c	PMID: 16428403	-4.41

34_C1	SMU_1843	*scrB*	Sucrose-6-phosphate hydrolase	Intergenic (-79/-117) *scrA* ← / → *scrB*		PMID: 9673256	-4.38
25_F5	SMU_1067c		ABC-2 membrane transporter family	V9D (GTT→GAT) *oppF*	SMU_436c		-4.18
25_E2	Intergenic between SMU_429c - SMU_431		Inner membrane protein; ATP-binding protein				-4.12
78 C12	Intergenic between SMU_530c - SMU_531c		Uncharacterized membrane protein; chorismate mutase	R239C (CGC→TGC) SMU_485			-4.08
09_G3	SMU_1577c		YobI family P-loop NTPase	G62S (GGC→AGC) *comR*	SMU_436c		-3.75
52_H1	SMU_2128	*ilvD*	Putative dihydroxy-acid dehydratase	L57F (TTA→TTC) *comR*			-3.73
26_B3	Intergenic between SMU_1192-SMU_1193	*dnaE* - *stsR*	DNA-directed DNA polymerase; transcriptional regulator	A131A (GCT→GCG) *ftsZ*			-3.49
58_H10	SMU_242c	*hisJ*	Amino acid transport system	R198C (CGC→TGC) *liaF*			-3.33
57_C1	Intergenic between *gpsA* - SMU_325				SMU_436c		-3.31
92 D2	Intergenic between SMU_1958c - SMU_1960c	*levF* - *levE*	Fructose-specific component IIC; Fructose-specific component IIB	Intergenic (+353/-30) SMU_60 → / → *comR*	SMU_436c; SMU_565c–SMU_566c		-3.21

80_G8	Intergenic between SMU_2047 - SMU_2048	*ptsG* - SMU_2048	Glucose-specific IIABC component; hypothetical protein	A205E (GCA→GAA) *oppC*		PMID: 17277067	-2.87
92_C9	Intergenic between SMU_1658-SMU_1659c	*nrgA* - *rsmI*	Putative ammonium transporter; 16S rRNA (cytidine(1402)-2'-O)-methyltransferase		ΔTn*Smu1*		-2.61
61 A12	SMU_104		Glycosyl hydrolase family		SMU_436c		-2.54
70_B8	SMU_683		Archaeal DNA helicase HerA or a related bacterial ATPase				-2.40
71_A5	Intergenic between SMU_991-SMU_992		Putative ribonucleotide reductase; hypothetical protein	intergenic (-283/-8) *pbp3* ← / → SMU_255			-2.39
71_G11	SMU_426	*copA*	Copper-transporting ATPase			*copZ*; PMID: 27753272	-2.34
67 D12	SMU_1577c		YobI family P-loop NTPase	Intergenic (+353/-30) SMU_60 → / → *comR*	SMU_436c		-2.31
76 A1	SMU_2066c	*pgfM1*	Glycosyltransferase	C→A SMU_t15	SMU_436c	PMCID: PMC12041528	-2.28
52_C4	SMU_870	*fruR*		L40F (TTA→TTC) *oppB*	SMU_436c	PMID: 28821551	-2.18

73_F12	Intergenic between SMU_872-SMU_873	*fruI-metE*	Fructose-specific enzyme IIABC component; putative homocysteine methyltransferase	Intergenic (+353/-30)<br>SMU_60 → / → *comR*		PMID: 28821551	-2.14
73_D12	SMU_1708	*trkA*	Putative potassium uptake system protein	D148N (<u>G</u>AT→<u>A</u>AT) *comR*			-2.11
76_B6	Intergenic between SMU_39-SMU_40	SMU_39 - SMU_40	HXXEE domain-containing protein; RelE family toxin	G62S (<u>G</u>GC→<u>A</u>GC) *comR*			-2.01
73_H11	SMU_129	*adhC*	Putative dihydrolipoamide acetyltransferase	Intergenic (+353/-30)<br>SMU_60 → / → *comR*			-1.94
76_A3	Intergenic between SMU_63c-SMU_64	SMU_63c-*ruvB*	Amyloid protein; Holliday junction DNA helicase	A98T (<u>G</u>CT→<u>A</u>CT) SMU_284		PMCID: PMC5775903	-1.93
03_F6	SMU_1128	*ciaH*	Histidine kinase		SMU_436c	PMCID: PMC470703	-1.92
71_C4	SMU_36		Suppressor of fused (Sufu) domain protein	T61S (A<u>C</u>C→A<u>G</u>C) SMU_374			-1.90
76_B7	SMU_1803c	SMU_1803c	DUF4230 domain-containing protein	Intergenic (+353/-30)<br>SMU_60 → / → comR<br>V145I (<u>G</u>TC→<u>A</u>TC) *malG*			-1.90
74_D12	SMU_402	*pfl*	Pyruvate formate-lyase	Intergenic (+353/-30)<br>SMU_60 → / → *comR*			-1.88

34_D10	SMU_833	*rgpI*	Glycosyltransferase	G471R (GGA→AGA) *oppA*		PMID: 31554721; PMID: 30578260	-1.87
76_A7	Intergenic between SMU_661 - SMU_662			Intergenic (+353/-30) SMU_60 → / → *comR*; D170E (GAC→GAG) *pgk*	SMU_436c; SMU_565c–SMU_566c		-1.86
76_D9	SMU_1005	*gtfC*	Glucosyltransferase		ΔTn*Smu1*	PMID: 9467380	-1.81
74_E10	SMU_58		Hypothetical protein	A205E (GCA→GAA) *oppC*	SMU_565c–SMU_566c		-1.75
92_B7	SMU_906	*vltB*	Viologen transporter	R161W (CGG→TGG) *oppF*	ΔTn*Smu1*		-1.70
74_E5	SMU_526c		Putative transcriptional regulator	D148N (GAT→AAT) *comR* M663R (ATG→AGG) *relA*			-1.68
65_E9	Intergenic between SMU_634-SMU_635	*queA* -	Putative S-adenosylmethionine--tRNA ribosyltransferase-isomerase; Predicted Fe2+/Mn2+ transporter	Intergenic (+353/-30) SMU_60 → / → SMU_61 R392H (CGC→CAC) SMU_1120			-1.66
76_A8	SMU_1337c	*mubM*	Mutanobactin biosynthesis	A73S (GCT→TCT) *pepN* Coding (3972/4887 nt; (T)8→9) *mubC*		PMID: 20639370	-1.65

37_A2	Intergenic between SMU_1106c - SMU_1107c		Histidine phosphatase family protein; SGNH/GDSL hydrolase family protein		*mubD*–SMU_1407c	PMID: 20639370	-1.65
65_F9	SMU_174c		Nucleotidyltransferase domain-containing protein	Intergenic (+353/-30) SMU_60 → / → *comR*			-1.63
66 G4	Intergenic between SMU_279 - SMU_281		Hypothetical protein; hypothetical protein	Y129D (<u>T</u>AT→<u>G</u>AT) SMU_1120 Intergenic (-64/+555) *comX* ← / ← SMU_t59	SMU_436c		-1.57
67_F5	Intergenic between SMU_1279c - SMU_1280c			Intergenic (+353/-30) SMU_60 → / → *comR*	SMU_436c; SMU_565c–SMU_566c		-1.55
71_G10	SMU_1004	*gtfB*	Glucosyltransferase			PMID: 9467380	-1.55
71_B11	SMU_1280c		Alpha/beta hydrolase fold	Intergenic (+353/-30) SMU_60 → / → *comR*	SMU_436c		-1.50
67_C10	SMU_410	*brpA*	Biofilm regulatory protein A	intergenic (-283/-8) *pbp3* ← / → SMU_255	SMU_436c	PMID: 16585759	-1.47
73 H9	SMU_495	*gldA*	Glycerol dehydrogenase	G62S (<u>G</u>GC→<u>A</u>GC) *comR*	SMU_436c; SMU_565c–SMU_566c		-1.45
83_E10	SMU_2160		Bacterial membrane protein YfhO	Intergenic (+353/-30) SMU_60 → / → *comR*	SMU_436c		-1.44

66_A6	SMU_116	*lacD2*	Tagatose 1,6-aldolase			PMID: 20190045	-1.42
83_A4	SMU_1879	*manZ*	Mannose-specific component IID	Intergenic (+353/-30) SMU_60 → / → *comR*		PMID: 24928869	-1.31
71_B6	SMU_770c	*mntH*	Putative manganese transporter	Δ457 bp SMU_t13-SMU_t17 T17T (ACC→ACT) SMU_1875		PMID: 31915219	-1.31
74_F1	SMU_149		Putative transposase				-1.30
74 C5	Intergenic between SMU_774c - SMU_775c		HAD family hydrolase; LTA synthase family protein	Intergenic (+353/-30) SMU_60 → / → *comR*	SMU_436c		-1.25
03_E7	SMU_1521	*hisM*	Amino acid transport system		SMU_565c–SMU_566c		-1.20
67_D11	SMU_1262c		Hypothetical protein	P304L (CCG→CTG) *oppD* intergenic (-29/+24) SMU_t41← / ← SMU_t42	ΔTn*Smu1*		-1.12
67_E5	SMU_1340	*mubC*	Nonribosomal peptide synthetase		Δ*TnSmu1*; *mubC*; SMU_436c; SMU_565c-SMU566c		-1.10
71_E6	SMU_1421	*pdhC*	Pyruvate dehydrogenase complex	Intergenic (+353/-30) SMU_60 → / → *comR*			-1.01

†Predicted mutations are genetic changes, such as base substitutions, or insertions, that the breseq pipeline has confirmed with high statistical confidence.

*Missing coverage evidence highlights regions where there is a significant drop or total absence of read alignment to the reference genome.

Table S3 *gtfBC* missing coverage evidence from whole genome sequencing

Strain ID	Start	End	Size	Z-score
**02_C11**	952944–953801	958428–957577	3777–5485	-4.3
**07_F12**	954365	958616	4252	-2.6
**09_G3**	954446	959141	4696	-3.8
**10_G5**	952960–953801	958428–957594	3794–5469	-5.05
**27_H5**	952953–953801	958430–957589	3789–5478	-3.9
**27_H8**	953917–953969	958611–958522	4554–4695	-4.5
**31_B12**	952953–953801	958432–957589	3789–5480	-3.8
**32_C11**	952302	956993	4692	-2.4
**34_B10**	952751–952787	957475–957422	4636–4725	-3.5
**34_B9**	952958–953801	958428–957589	3789–5471	-3.4
**34_E10**	952749	957481	4733	-3.3
**34_F2**	953918	958574	4657	-3.5
**41_A4**	954196	958875–958750	4555–4680	-4.3
**49_B8**	952915–953801	956997	3197–4083	-5.2
**55_E6**	954431	959141	4711	-3.3
**58_F12**	954180–954216	958877–958793	4578–4698	-2.4
**61_F7**	952951–953801	958428–957571	3771–5478	-2.7
**81_E6**	952953–953801	958424–957573	3773–5472	-3.1

**90_A4**	954201–954206	958869–958762	4557–4669	-6.1
**90_H10**	952147	956841	4694	-4.2

Table S4 Whole genome sequencing of transposon insertions mutants with evidence of *gtfBC* recombination and major biofilm defects

Strain ID	Location of Tn insertion	Gene name	Gene description	Predicted mutations†	Missing coverage evidence*
**02_C11**	SMU_545	-	Hypothetical protein	I591I (ATC→ATT) *gtfB*	*gtfBC*
**07_F12**	SMU_1657c	*glnB*	Nitrogen regulatory protein PII	Y1084N (TAC→AAC) *gtfB*<br>L964F (CTC→TTT) *gtfC*<br>L964F (CTC→TTT) *gtfC*	*gtfBC; bacD*–SMU_1408c
**09_G3**	SMU_910	*gtfD*	Glucosyltransferase	A295D (GCT→GAT) *oppD*<br>A1145V (GCT→GTT) *gtfC*	*gtfBC*
**10_G5**	SMU_1243	-	Bacterial low temperature requirement A protein	-	*gtfBC*; SMU_436c; SMU_565c–SMU_566c
**27_H5**	SMU_1337c	*mubM*	Mutanobactin biosynthesis	-	*gtfBC*
**27_H8**	SMU_155	*pnpA*	Polyribonucleotide nucleotidyltransferase	R74S (CGT→AGT) *oppA*<br>L964F (CTC→TTT) *gtfC*<br>L964F (CTC→TTT) *gtfC*<br>T331T (ACA→ACC) *gyrB*<br>Y236C (TAT→TGT) SMU_1950<br>I3I (ATA→ATT) SMU_1966c	*gtfBC*
**31_B12**	SMU_167	-	Hypothetical protein	A185D (GCT→GAT) *oppD*	*gtfBC*
**32_C11**	SMU_78	*fruA*	Fructan hydrolase	R394R (AGA→AGG) *gtfB*<br>Intergenic (-151/+509) *queG* ← / ← SMU_1329c<br>Intergenic (-204/-111) SMU_1397c ← / → *irvR*	*gtfBC*
**34_B10**	SMU_728	-	Putative oxidoreductase	Intergenic (+353/-30) SMU_60 → / → *comR*<br>V61F (GTT→TTT) *pfs*	*gtfBC*

**34_B9**	SMU_305 - SMU_306	-	Pseudogene	Coding (4/1110 nt) $(T)_{8\rightarrow7}$ SMU_1432c	*gtfBC*
**34_E10**	SMU_728	-	Putative oxidoreductase	Intergenic (+353/-30), SMU_60 → / → *comR*<br>V61F (GTT→TTT) SMU_1632	*gtfBC*
**34_F2**	SMU_2094c	-	Conserved hypothetical protein	R198C (CGC→TGC) *liaF*<br>L964F (CTC→TTT) *gtfC*<br>L964F (CTC→TTT) *gtfC*<br>G231G (GGC→GGA) SMU_1936c	*gtfBC*
**41_A4**	SMU_1572	*murZ*		-	*gtfBC*
**49_B8**	SMU_1157c	-	SIR2 family NAD-dependent protein deacylase	Intergenic (+353/-30), SMU_60 → / → *comR*<br>T400T (ACT→ACC) *gtfB*<br>I401I (ATC→ATT) *gtfB*<br>S585S (TCA→TCT) *gtfB*<br>T587T (ACT→ACC) *gtfB*<br>I591I (ATC→ATT) *gtfB*	*gtfBC*
**55_E6**	Intergenic between SMU_909 - SMU_910	SMU_909 - *gtfD*	Impacting the expression of *gtfD*	G471R (GGA→AGA), *oppA*<br>A1145V (GCT→GTT) *gtfC*	SMU_565c–SMU_566c; *gtfBC*
**58_F12**	SMU_1436c	-	Hypothetical protein	-	*gtfBC*
**61_F7**	SMU_515	-	Oleate hydratase	A185D (GCT→GAT) *oppD*	*gtfBC*
**81_E6**	SMU_1321c	-	ATP-grasp domain-containing protein	V168A (GTT→GCT) SMU_252<br>A279D (GCT→GAT) *oppA*<br>*G134R (GGT→CGT)* ung	*gtfBC*
**90_A4**	SMU_1116c - SMU_1117	SMU_1116c - *naoX*	DUF4391 domain-containing protein; NADH oxidase	T1067T (ACC→ACT) *gtfC*	*gtfBC*

**90_H10**	SMU_1398	*irvR*	LexA-like repressor protein	-	*gtfBC*

†Predicted mutations are genetic changes, such as base substitutions, or insertions, that the breseq pipeline has confirmed with high statistical confidence.

*Missing coverage evidence highlights regions where there is a significant drop or total absence of read alignment to the reference genome.

Table S5 Strains used in this study

Strain	Description	Source
S. mutans strains		
UA159	Wild-type	Shields Lab
LKM68	Tn*Smu1* replaced with a spectinomycin marker	(1)
Δ*gtfB*	Tn insertion within *gtfB* gene	This study
ΔSMU_635	SMU_635 replaced with a kanamycin marker	This study
ΔSMU_1803c	SMU_1803c replaced with a kanamycin marker	This study
ΔSMU_2160	SMU_2160 replaced with a kanamycin marker	This study

S6 Oligonucleotides used in this study

Oligonucleotide Name	Sequence (5' - 3')	Experiment
*gtfBC*_F	tcagttttaagaggacggcac	*gtfBC* verification
*gtfBC*_R	agcctgagaaatttacagctca	*gtfBC* verification
SMU_635_A	ctcccattgatctccctgaa	Knockout
SMU_635_B	atgcggatccgactcctgcgaccgaaataa	Knockout
SMU_635_C	atgcggatcctgatagtgggactattaaccatgc	Knockout
SMU_635_D	tatcatcggctgacaaacca	Knockout
SMU_2160_A	gtcctatcaggtggcgaaaa	Knockout
SMU_2160_B	atgcggatcctaaaaccaagcccagcctta	Knockout
SMU_2160_C	atgcggatccaatgacttttgtgccccaag	Knockout
SMU_2160_D	cgttggtcaaggggttaaga	Knockout
SMU_1803c_A	atttcccgcattgttgtctc	Knockout
SMU_1803c_B	atgcggatccgctatcagtgcagcaaacca	Knockout
SMU_1803c_C	atgcggatccggtaaagctgccacatcgtt	Knockout
SMU_1803c_D	ccacgactaccggcatatct	Knockout
SMU_21_F	tttcgttaaaccagcggtaga	Library PCR verification
SMU_21_R	tgctttaacatcgcctttca	Library PCR verification
SMU_71_F	gtcacacaggcaggaggaat	Library PCR verification
SMU_71_R	accaaacacccgctaaacca	Library PCR verification
SMU_91_F	ttaacagcaccaggtttccg	Library PCR verification
SMU_91_R	atcgcacgttggacctcatc	Library PCR verification
SMU_148_F	ggcatctgatttcacacgtcc	Library PCR verification
SMU_148_R	cctttgcgttcacataagtccc	Library PCR verification
SMU_270_F	tggtacaacaagctgcgaca	Library PCR verification
SMU_270_R	gccaaatcctgccagaccta	Library PCR verification
SMU_333_F	tggaatacagaaaaagcggtgac	Library PCR verification
SMU_333_R	ccaagacataagattcaattgctcca	Library PCR verification
SMU_426_F	atatgggggttgaagtggcc	Library PCR verification
SMU_426_R	tccctctttgtgaccatctga	Library PCR verification

SMU_510c_F	gccagcactatctcctagcc	Library PCR verification
SMU_510c_R	ggcaattttcccatcgagcg	Library PCR verification
SMU_682_F	tcagaagttatcggggctgc	Library PCR verification
SMU_682_R	tgcgatggaaaactaaagagatgt	Library PCR verification
SMU_832_F	ttagcccaactttcgcagct	Library PCR verification
SMU_832_R	gcactccgggaaaaataacgataa	Library PCR verification
SMU_999_F	attggtcaggatgcttctgc	Library PCR verification
SMU_999_R	acagccatatcacgaagagaca	Library PCR verification
SMU_1206c_F	gctacaccctctatcacaccg	Library PCR verification
SMU_1206c_R	aacttctctgggagcaactc	Library PCR verification
SMU_1237c_F	tcttgagcagaatacagttctttga	Library PCR verification
SMU_1237c_R	acttttgaatcgcttgcacaac	Library PCR verification
SMU_1363c_F	tggcacaaaaggcacatagt	Library PCR verification
SMU_1363c_R	ccgtcccaacaactcctcaa	Library PCR verification
SMU_1450_F	acgcttcaaaatcggaacact	Library PCR verification
SMU_1450_R	gcatgtacgtatgattccgcg	Library PCR verification
SMU_1577c_F	tggttttatcgctgagttgct	Library PCR verification
SMU_1577c_R	tcgaaccatattcttgttcctgt	Library PCR verification
SMU_1772c_F	tgacaaacaaagactagattcaaatgt	Library PCR verification
SMU_1772c_R	tggattgtatttaggtgaaagttgtgt	Library PCR verification
SMU_1916_F	tctcccaccgcattgaatca	Library PCR verification
SMU_1916_R	ttgaacacactgagaaagaggt	Library PCR verification
SMU_2059c_F	tgttttgctcggttttggct	Library PCR verification
SMU_2059c_R	agagatgttgcatcccaagact	Library PCR verification
SMU_2127_F	cgtattcaaggggtggcctt	Library PCR verification
SMU_2127_R	tgaccgcttcctttcatcaca	Library PCR verification

Scripts

HiPerGator Command Line
Split by barcodes

```
#SBATCH --array=1-20
#SBATCH --job-name=bar#split
#SBATCH --partition=hpg-default
#SBATCH --account=robert.shields
#SBATCH --mail-user=anasolanomorales@ufl.edu
#SBATCH --mail-type=FAIL
#SBATCH --output stdout_%A_%a_barcodesplit.out
#SBATCH --nodes=1
#SBATCH --ntasks=1
#SBATCH --cpus-per-task=1
#SBATCH --mem=8gb
#SBATCH --time=04:00:00
### Timestamp
start=`date +%s`
### Changing directory
cd /blue/robert.shields/anasolanomorales/Tn_seq/samples
### Variable creation
sampleID=$( cat ../barcodes.txt | sed -n "${SLURM_ARRAY_TASK_ID},${SLURM_ARRAY_TASK_ID}p" | cut -
f 1 )
barcode=$( cat ../barcodes.txt | sed -n "${SLURM_ARRAY_TASK_ID},${SLURM_ARRAY_TASK_ID}p" | cut -f
2 )
echo -e ${SLURM_ARRAY_TASK_ID}"\t"${sampleID}"\t"${barcode}
### Samples splitting
echo "Splitting fastq file"
zcat /blue/robert.shields/anasolanomorales/Tn_seq/fastq/Tn_1_S0_R1_001.fastq.gz | tr ' ' '_' | paste - - - - |
awk -v sample=${sampleID} -v code=${barcode} '{ if( substr($2,1,8)==code ) sum=sum+1 ; if(
substr($2,1,8)==code ) print "@"sample"_"code"_"sum"\n"substr($2,9,16)"\n"$3"\n"substr($4,9,16) }' >
${sampleID}.fastq
### Compressing fastq2gz
echo "fastq2gz"
gzip ${sampleID}.fastq
### Counting reads
count=$( zcat ${sampleID}.fastq.gz | tail -4 | head -1 | tr '_' '\t' | cut -f 3 )
echo -e "Reads count = "${count}
### Print time
end=`date +%s`
runtime=$((end-start))
out=$(echo "scale=2; (${runtime}/1)" | bc); echo "${out} seconds"
```

FastQC Pair-end

```
#!/bin/bash
#SBATCH --array=1-40
#SBATCH --job-name=fastqc
#SBATCH --partition=hpg-default
#SBATCH --account=fgibson
#SBATCH --qos=fgibson-b
#SBATCH --mail-user=ariverosw@ufl.edu
#SBATCH --mail-type=FAIL
#SBATCH --output stdout_%A_%a_fastqc.out
#SBATCH --nodes=1
#SBATCH --ntasks=1
#SBATCH --cpus-per-task=24
#SBATCH --mem=8gb
#SBATCH --time=03:00:00
start=`date +%s`
### Changing directory
cd /blue/robert.shields/arwalker/fastqc
### SampleID
sampleID=$( eval cat ../samples.txt | sed -n "${SLURM_ARRAY_TASK_ID},${SLURM_ARRAY_TASK_ID}p" |
cut -f 1)
#sampleID=${SLURM_ARRAY_TASK_ID}
### Running FASTQC
ml fastqc
fastqc -t ${SLURM_CPUS_PER_TASK}
/blue/robert.shields/anasolanomorales/tn_seq/samples/${sampleID}.fastq.gz --outdir ./
### Print time
end=`date +%s`
runtime=$((end-start))
out=$(echo "scale=2; (${runtime}/1)" | bc); echo "${out} seconds"
```

Consolidating images pair-end

```
#!/usr/bin/bash
scripts=${PWD}
cd /blue/robert.shields/arwalker/fastqc
mkdir images
echo "<!DOCTYPE html>" > index.html
echo "<html>" >> index.html
echo "<head>" >> index.html
echo "<meta charset="utf-8">" >> index.html
echo "<title>UA159 Tn-Seq Analysis</title>" >> index.html
echo "</head>" >> index.html
echo "<body>" >> index.html
for i in $(cat ../samples.txt | cut -f 1 )
  do
    unzip -p ../fastqc/${i}_fastqc.zip ${i}_fastqc/Images/per_base_quality.png > ./images/${i}.png
    echo -e "<p>"${i}"</p>" >> index.html
    echo -e "<figure class="half" style="display:flex">\"" >> index.html
    echo -e "<img style="width:600px" src= \"./images/${i}.png\" >" >> index.html
    echo -e "</figure>" >> index.html
done
echo "</body>" >> index.html
echo "</html>" >> index.html
cat index.html | sed 's\<figure class=half style=display:flex>"\<figure class=half style=display:flex>\g' >
temp.html
mv temp.html samples_fastqc.html
rm index.html
cd ${scripts}
```

Mapping

```
#!/usr/bin/bash
#SBATCH --array=1-40
#SBATCH --job-name=mapping
#SBATCH --partition=hpg-default
#SBATCH --account=robert.shields
#SBATCH --mail-user=anasolanomorales@ufl.edu
#SBATCH --mail-type=FAIL
#SBATCH --output stdout_%A_%a_mapping.out
#SBATCH --nodes=1
#SBATCH --ntasks=1
#SBATCH --cpus-per-task=1
#SBATCH --mem=8gb
#SBATCH --time=04:00:00
### Timestamp
start=`date +%s`
### Changing directory
cd /blue/robert.shields/anasolanomorales/tn_seq/mapping
### Variable creation
sampleID=$( cat ../samples.txt | sed -n "${SLURM_ARRAY_TASK_ID},${SLURM_ARRAY_TASK_ID}p" | cut -f
1 )
echo -e ${SLURM_ARRAY_TASK_ID}"\t"${sampleID}
### Mapping
echo "Mapping fastq file"
ml bwa
bwa aln /blue/robert.shields/anasolanomorales/references/UA159.fasta ../samples/${sampleID}.fastq.gz >
${sampleID}_raw_aln.sai
bwa samse /blue/robert.shields/anasolanomorales/references/UA159.fasta ${sampleID}_raw_aln.sai
../samples/${sampleID}.fastq.gz > ${sampleID}_raw_aln.sam
ml purge
### SAM-BAM
echo "Converting SAM-BAM"
ml samtools
samtools view -b ${sampleID}_raw_aln.sam > ${sampleID}_raw_aln.bam
samtools sort ${sampleID}_raw_aln.bam -o ${sampleID}_raw_aln_sorted.bam
ml purge
###Remove intermediate files
echo "Removing intermediate files"
rm ${sampleID}_raw_aln.sai
rm ${sampleID}_raw_aln.sam
rm ${sampleID}_raw_aln.bam
### Print time
end=`date +%s`
runtime=$((end-start))
out=$(echo "scale=2; (${runtime}/1)" | bc); echo "${out} seconds"
```

Filtering TA insertions

```
#!/usr/bin/bash
#SBATCH --array=1-40
#SBATCH --job-name=filtering
#SBATCH --partition=hpg-default
#SBATCH --account=robert.shields
#SBATCH --mail-user=ariverosw@ufl.edu
#SBATCH --mail-type=FAIL
#SBATCH --output stdout_%A_%a_filtering.out
#SBATCH --nodes=1
#SBATCH --ntasks=1
#SBATCH --cpus-per-task=1
#SBATCH --mem=8gb
#SBATCH --time=04:00:00
### Timestamp
start=`date +%s`
### Changing directory
cd /blue/robert.shields/arwalker/ta_insertions
### Variable creation
sampleID=$( cat ../samples2.txt | sed -n "${SLURM_ARRAY_TASK_ID},${SLURM_ARRAY_TASK_ID}p" | cut
-f 1 )
echo -e ${SLURM_ARRAY_TASK_ID}"\t"${sampleID}
### Samtools process
echo "Filtering BAM file"
ml samtools
### Indexing BAM file
samtools index ../mapping/${sampleID}_raw_aln_sorted.bam
### TA sites coverage
samtools depth -aa -b ../UA159/UA159_TA_Abase_1bp.bed ../mapping/${sampleID}_raw_aln_sorted.bam >
${sampleID}_raw_aln_sorted.TA.depth.tsv
ml purge
### Print time
end=`date +%s`
runtime=$((end-start))
out=$(echo "scale=2; (${runtime}/1)" | bc); echo "${out} seconds"
```

Annotation table

```
#!/usr/bin/bash
scriptPWD=${PWD}
cd /blue/robert.shields/anasolanomorales/references
### Create annotation table
awk -F '\t' '
$3 == "CDS" {
	locus_tag = name = annotation = ".";
	split($9, attrs, ";");
	for (i in attrs) {
	if (attrs[i] ~ /locus_tag=/) {
	split(attrs[i], x, "="); locus_tag = x[2];
	} else if (attrs[i] ~ /^gene=/) {
	split(attrs[i], x, "="); name = x[2];
	} else if (attrs[i] ~ /^product=/ || attrs[i] ~ /^Note=/) {
	split(attrs[i], x, "="); annotation = x[2];
	}
	}
	# fallback: if no gene= was found, use locus_tag
	if (name == ".") {
	name = locus_tag;
	}
	start = $4;
	end = $5;
	strand = $7;
	gene_length = end - start + 1;
	print locus_tag "\t" name "\t" start "\t" end "\t" gene_length "\t" strand "\t" annotation;
}' /blue/robert.shields/anasolanomorales/references/UA159.gff > UA159_cds_annotations.tsv
cd ${scriptPWD}
```

RStudio
Data counts analysis plotting

```
### This script will write several filtered dataset at different filtering parameters
### These were later plotted by script 02_plotting_counts_threshold.R
require(dplyr)
require(tidyr)
require(stringr)
require(purrr)
require(openxlsx)

setwd("~/UF/Dental/rob/ana/")

samples <- read.delim("samples.txt", header=F)
colnames(samples) <- "locus_tag"

data.counts <- read.delim("./counts/htseq_counts_all_samples.txt", header=F, sep= ' ')
colnames(data.counts) <- c("locus_tag", samples[,1])
data.counts <- data.counts[, c(1, 2:9, 13:21, 10:12, 34:41, 25:33, 22:24)]
data.counts <- data.counts[-which(substr(data.counts[,1],1,2)=="__"),]

### ---------------------------
### 1. Define the factor levels that exist in YOUR data
###    (based on your column names and what we inspected)
### ---------------------------

# X group (8 levels)
X_levels  <- c("XA","XB","XC","XD","XE","XF","XG","XH")

# Y group (12 levels)
Y_levels  <- c("Y1","Y2","Y3","Y4","Y5","Y6","Y7","Y8","Y9","Y10","Y11","Y12")

# ZL = Z letter group (8 levels)
ZL_levels <- c("ZA","ZB","ZC","ZD","ZE","ZF","ZG","ZH")

# ZN = Z number group
# Important: "Z4" and "Z5" are merged in your file header as "Z4Z5".
# So right now we treat "Z4Z5" as a single observed condition.
# That gives us 11 total unique ZN conditions.
ZN_levels <- c("Z1","Z2","Z3","Z4","Z5","Z6","Z7","Z8","Z9","Z10","Z11","Z12")

### ---------------------------
### 2. Build the full combination grid
###    This gives all combos of:
###    X (8) x Y (12) x ZL (8) x ZN (11) = 8448 rows
### ---------------------------
all_combos <- expand.grid(
  X  = X_levels,
  Y  = Y_levels,
  ZL = ZL_levels,
  ZN = ZN_levels,
  KEEP.OUT.ATTRS = FALSE,
  stringsAsFactors = FALSE
)

### ---------------------------
```

```
### 3. Reshape your raw data.counts into long format
###    Assumptions:
###    - data.counts exists in your environment
###    - first column is locus_tag (gene ID like "SMU_1006")
###    - remaining columns are the condition columns:
###        XA, XB, ..., XH,
###        Y1...Y12,
###        ZA...ZH,
###        Z1, Z2, Z3, Z4Z5, Z6...Z12
### ---------------------------

counts_long <- data.counts %>%
  pivot_longer(
    cols      = -locus_tag,
    names_to  = "condition",
    values_to = "count"
  ) %>%
  mutate(
    # group = first letter of condition ("X","Y","Z")
    group = str_sub(condition, 1, 1),

    # We'll label which subgroup each condition belongs to:
    subgroup = dplyr::case_when(
      condition %in% X_levels      ~ "X",
      condition %in% Y_levels      ~ "Y",
      condition %in% ZL_levels     ~ "ZL",
      condition %in% ZN_levels     ~ "ZN",
      TRUE                         ~ "OTHER"   # safety net
    )
  )

# After this step, counts_long looks like:
# locus_tag | condition | count | group | subgroup
# SMU_1006  | XA        | 269   | "X"   | "X"
# SMU_1006  | Y10       | 111474| "Y"   | "Y"
# SMU_1006  | ZA        | 137   | "Z"   | "ZL"
# SMU_1006  | Z10       | 48600 | "Z"   | "ZN"
# etc.

### ---------------------------
### 4. Define a helper that, for ONE gene, attaches its observed counts
###    to all 8*12*8*11 = 8448 theoretical combinations
### ---------------------------

expand_gene <- function(gene_df) {

  gene_id <- unique(gene_df$locus_tag)

  # Build lookup tables for each subgroup (X, Y, ZL, ZN)
  x_lookup <- gene_df %>%
    filter(subgroup == "X") %>%
    select(condition, count)

  y_lookup <- gene_df %>%
    filter(subgroup == "Y") %>%
    select(condition, count)
```

```
  zl_lookup <- gene_df %>%
    filter(subgroup == "ZL") %>%
    select(condition, count)

  zn_lookup <- gene_df %>%
    filter(subgroup == "ZN") %>%
    select(condition, count)

  # Join these lookups into the full combo grid
  gene_expanded <- all_combos %>%
    # Attach X_count for that gene
    left_join(x_lookup,  by = c("X"  = "condition")) %>%
    rename(X_count = count) %>%
    # Attach Y_count
    left_join(y_lookup,  by = c("Y"  = "condition")) %>%
    rename(Y_count = count) %>%
    # Attach ZL_count
    left_join(zl_lookup, by = c("ZL" = "condition")) %>%
    rename(ZL_count = count) %>%
    # Attach ZN_count
    left_join(zn_lookup, by = c("ZN" = "condition")) %>%
    rename(ZN_count = count) %>%
    # Add gene ID and placeholder for a true joint measurement
    mutate(
      locus_tag   = gene_id,
      joint_count = NA_real_
    ) %>%
    select(
      locus_tag,
      X, Y, ZL, ZN,
      X_count, Y_count, ZL_count, ZN_count,
      joint_count
    )

  # Explanation:
  # - X_count is this gene's observed count in that X condition alone.
  # - Y_count is this gene's observed count in that Y condition alone.
  # - ZL_count is this gene's observed count in that Z-letter condition alone.
  # - ZN_count is this gene's observed count in that Z-number condition alone.
  # - joint_count stays NA because you do NOT have direct experimental data
  #   for that exact (X,Y,ZL,ZN) four-way combination.

  return(gene_expanded)
}

### ---------------------------
### 5. Apply that helper to every gene
###    Result: one giant data frame with 8448 rows per gene
### ---------------------------

expanded_all_genes <- counts_long %>%
  group_by(locus_tag) %>%
  group_split() %>%
  map_df(expand_gene)
```

```
### ---------------------------
### 6. Quick QC examples
### ---------------------------

# How many unique combos per gene?
expanded_all_genes %>%
  filter(locus_tag == first(locus_tag)) %>%
  nrow()
# Expect 8448 with current levels (8 * 12 * 8 * 11)

# Peek at one gene, first few rows:
expanded_all_genes %>%
  filter(locus_tag == first(locus_tag)) %>%
  head()

# Check that we didn't lose anything weird
summary(expanded_all_genes)

###
### 7.
###

# Choose a similarity tolerance
# Example: CV < 0.2 (20% variability)
cv_threshold <- 0.2
count_threshold <- 500

expanded_all_genes <- expanded_all_genes %>%
  rowwise() %>%
  mutate(
    # compute mean and sd across the four counts
    mean_count = mean(c(X_count, Y_count, ZL_count, ZN_count), na.rm = TRUE),
    sd_count   = sd(c(X_count, Y_count, ZL_count, ZN_count), na.rm = TRUE),

    # coefficient of variation
    cv = ifelse(mean_count > 0, sd_count / mean_count, NA_real_),

    # TRUE/FALSE if all 4 are within the allowed CV
    similar_by_cv = !is.na(cv) & cv <= cv_threshold,

    # also check absolute range if you prefer Option A:
    range_count = max(c(X_count, Y_count, ZL_count, ZN_count), na.rm = TRUE) -
      min(c(X_count, Y_count, ZL_count, ZN_count), na.rm = TRUE),
    similar_by_range = range_count <= cv_threshold * mean_count  # ≤ 20 % spread
  ) %>%
  ungroup()

for (cv_threshold in c(0.05,0.1,0.15,0.2,0.25)) {

  for (count_threshold in c(500,1000,1500,2000,2500)) {

    expanded_all_genes2 <- expanded_all_genes[expanded_all_genes[,11]>count_threshold &
expanded_all_genes[,13]<cv_threshold,]
    write.xlsx(expanded_all_genes2, paste0("./output/ta_insertions_per_gene_cv-",cv_threshold,"_counts-
```

```
",count_threshold,"_npositions_",nrow(expanded_all_genes2),".xlsx") )
  }
}
```

Plotting counts threshold

```
library(openxlsx)
library(dplyr)

setwd("~/UF/Dental/rob/ana/")

# ------------------------------------------------------------
# 1. Load original data
# ------------------------------------------------------------
origin <- read.xlsx("./ta_insertions_annotated_all_samples.xlsx", sheet = "Genes")

# Fix column names as you did
colnames(origin)[c(2,3)] <- c("pos1","pos2")
colnames(origin)[c(6,7)] <- c("start","end")

# Make sure geneID column is named correctly (assuming it is column 4 already)
# str(origin) to confirm if needed

# ------------------------------------------------------------
# 2. Define column indices for each group
# ------------------------------------------------------------
# Based on your previous script:
# 11:18 -> X letters
# 19:30 -> Y numbers
# 31:38 -> Z letters
# 39:50 -> Z numbers

idx_X  <- 11:18
idx_Y  <- 19:30
idx_ZL <- 31:38
idx_ZN <- 39:50

# ------------------------------------------------------------
# 3. Thresholds to test
# ------------------------------------------------------------
thresholds <- c(100, 250, 500, 750, 1000, 1250, 1500,
                1750, 2000, 3000, 4000, 5000)

# ------------------------------------------------------------
# 4. Function to compute summary at one threshold
# ------------------------------------------------------------
summarize_at_threshold <- function(thr, dat = origin) {

  # group-wise counts > threshold, vectorized with rowSums
  g1 <- rowSums(dat[, idx_X]  > thr, na.rm = TRUE)  # X letters
  g2 <- rowSums(dat[, idx_Y]  > thr, na.rm = TRUE)  # Y numbers
  g3 <- rowSums(dat[, idx_ZL] > thr, na.rm = TRUE)  # Z letters
  g4 <- rowSums(dat[, idx_ZN] > thr, na.rm = TRUE)  # Z numbers

  # 4-group rule: exactly 1 in each group
  check4 <- (g1 == 1 & g2 == 1 & g3 == 1 & g4 == 1)

  # 5-group rule: total 5, but each group has at least 1
  check5 <- ((g1 + g2 + g3 + g4) == 5 &
             g1 >= 1 & g2 >= 1 & g3 >= 1 & g4 >= 1)
```

```
  # Subsets
  data4 <- dat[check4, , drop = FALSE]
  data5 <- dat[check5, , drop = FALSE]

  # Unique gene counts (assuming geneID column is named "geneID")
  n_genes4 <- length(unique(data4$geneID))
  n_genes5 <- length(unique(data5$geneID))

  tibble(
    threshold     = thr,
    n_insertions4 = sum(check4),
    n_genes4      = n_genes4,
    n_insertions5 = sum(check5),
    n_genes5      = n_genes5
  )
}

# ------------------------------------------------------------
# 5. Run over all thresholds
# ------------------------------------------------------------
summary_thr <- bind_rows(lapply(thresholds, summarize_at_threshold))

print(summary_thr)

# Optionally save
write.table(
  summary_thr,
  file      = "./data.ta/threshold_sweep_summary.tsv",
  sep       = "\t",
  quote     = FALSE,
  row.names = FALSE
)

## ----------------------------------------------------------------
##  Add elbow-detection and pretty TIFF figure
## ----------------------------------------------------------------

library(ggplot2)
library(tidyr)
library(patchwork)

# ---------- 1) Elbow detection (Kneedle-style) ----------
detect_elbow <- function(x, y) {
  x_scaled <- (x - min(x)) / (max(x) - min(x))
  y_scaled <- (y - min(y)) / (max(y) - min(y))
  p1 <- c(x_scaled[1], y_scaled[1])
  p2 <- c(x_scaled[length(x_scaled)], y_scaled[length(y_scaled)])
  distances <- sapply(seq_along(x_scaled), function(i) {
    p <- c(x_scaled[i], y_scaled[i])
    abs(det(rbind(p2 - p1, p - p1))) / sqrt(sum((p2 - p1)^2))
  })
  which.max(distances)
}

idx_insert <- detect_elbow(summary_thr$threshold, summary_thr$n_insertions4)
```

```
idx_genes  <- detect_elbow(summary_thr$threshold, summary_thr$n_genes4)

elbow_insert <- summary_thr$threshold[idx_insert]
elbow_genes  <- summary_thr$threshold[idx_genes]

cat("Suggested elbow for insertions (4-group):", elbow_insert, "\n")
cat("Suggested elbow for genes (4-group)     :", elbow_genes,  "\n")

# ---------- 2) Long format for ggplot ----------
df_long_ins <- summary_thr %>%
  pivot_longer(cols = c("n_insertions4","n_insertions5"),
               names_to = "group",
               values_to = "n_insertions") %>%
  mutate(group = ifelse(group == "n_insertions4", "4-group", "5-group"))

df_long_genes <- summary_thr %>%
  pivot_longer(cols = c("n_genes4","n_genes5"),
               names_to = "group",
               values_to = "n_genes") %>%
  mutate(group = ifelse(group == "n_genes4", "4-group", "5-group"))

group_colors <- c("4-group" = "#1f78b4", "5-group" = "#e31a1c")

# ---------- 3) Insertions plot ----------
p_ins <- ggplot(df_long_ins,
                aes(x = threshold, y = n_insertions, color = group)) +
  geom_line(linewidth = 1) +
  geom_point(size = 3) +
  geom_vline(xintercept = elbow_insert, linetype = "dashed", color = "black") +
  annotate("label", x = elbow_insert,
           y = max(df_long_ins$n_insertions) * 0.9,
           label = paste("Elbow =", elbow_insert),
           size = 3, label.size = 0.2, fill = "white") +
  scale_color_manual(values = group_colors) +
  scale_x_continuous(breaks = summary_thr$threshold) +
  theme_bw(base_size = 13) +
  theme(
    legend.position = "top",
    legend.title = element_blank(),
    plot.title = element_text(face = "bold"),
    axis.text.x = element_text(angle = 45, hjust = 1)
  ) +
  labs(
    title = "TA insertions retained vs. count threshold",
    x = "Threshold (> X counts)",
    y = "Number of TA insertions"
  )

# ---------- 4) Genes plot (rotated x labels) ----------
p_genes <- ggplot(df_long_genes,
                  aes(x = threshold, y = n_genes, color = group)) +
  geom_line(linewidth = 1) +
  geom_point(size = 3) +
  geom_vline(xintercept = elbow_genes, linetype = "dashed", color = "black") +
  annotate("label", x = elbow_genes,
           y = max(df_long_genes$n_genes) * 0.925,
```

```
      label = paste("Elbow =", elbow_genes),
      size = 3, label.size = 0.2, fill = "white") +
  scale_color_manual(values = group_colors) +
  scale_x_continuous(breaks = summary_thr$threshold) +
  theme_bw(base_size = 13) +
  theme(
    legend.position = "top",
    legend.title = element_blank(),
    plot.title = element_text(face = "bold"),
    axis.text.x = element_text(angle = 45, hjust = 1)
  ) +
  labs(
    title = "Unique genes represented vs. count threshold",
    x = "Threshold (> X counts)",
    y = "Number of genes"
  )

# ---------- 5) Combine and export ----------
combined_plot <- p_ins / p_genes +
  plot_layout(heights = c(1, 1)) +
  plot_annotation(
    title = "Threshold sensitivity analysis for pooled Tn-Seq selection",
    theme = theme(
      plot.title = element_text(face = "bold", hjust = 0.5, size = 16),
      legend.position = "top",
      legend.title = element_blank()
    )
  )

print(combined_plot)

# Save as TIFF (high resolution)
tiff("./data.ta/threshold_sweep_pretty.tiff",
     width = 8, height = 8, units = "in", res = 600)
print(combined_plot)
dev.off()

### Manuscript-friendly wording
### "Sensitivity analysis across dynamic count thresholds (100–5000)
### showed a sharp decline in gene representation beyond ~1000 reads.
### Therefore, a threshold of >500 reads was selected to balance library
### representativeness and robustness, with high-confidence insertions
### identified using a geometric mean score across the four pooling dimensions."
```

TA insertions across samples

```
library(openxlsx)
library(dplyr)

setwd("~/UF/Dental/rob/ana/")

# -----------------------------------------------------------
# 1. Load original data
# -----------------------------------------------------------
origin <- read.xlsx("./ta_insertions_annotated_all_samples.xlsx", sheet = "Genes")

# Fix column names as you did
colnames(origin)[c(2,3)] <- c("pos1","pos2")
colnames(origin)[c(6,7)] <- c("start","end")

# Make sure geneID column is named correctly (assuming it is column 4 already)
# str(origin) to confirm if needed

# -----------------------------------------------------------
# 2. Define column indices for each group
# -----------------------------------------------------------
# Based on your previous script:
# 11:18 -> X letters
# 19:30 -> Y numbers
# 31:38 -> Z letters
# 39:50 -> Z numbers

idx_X  <- 11:18
idx_Y  <- 19:30
idx_ZL <- 31:38
idx_ZN <- 39:50

# -----------------------------------------------------------
# 3. Thresholds to test
# -----------------------------------------------------------
thresholds <- c(100, 250, 500, 750, 1000, 1250, 1500,
                1750, 2000, 3000, 4000, 5000)

# -----------------------------------------------------------
# 4. Function to compute summary at one threshold
# -----------------------------------------------------------
summarize_at_threshold <- function(thr, dat = origin) {

  # group-wise counts > threshold, vectorized with rowSums
  g1 <- rowSums(dat[, idx_X]  > thr, na.rm = TRUE)  # X letters
  g2 <- rowSums(dat[, idx_Y]  > thr, na.rm = TRUE)  # Y numbers
  g3 <- rowSums(dat[, idx_ZL] > thr, na.rm = TRUE)  # Z letters
  g4 <- rowSums(dat[, idx_ZN] > thr, na.rm = TRUE)  # Z numbers

  # 4-group rule: exactly 1 in each group
  check4 <- (g1 == 1 & g2 == 1 & g3 == 1 & g4 == 1)

  # 5-group rule: total 5, but each group has at least 1
  check5 <- ((g1 + g2 + g3 + g4) == 5 &
             g1 >= 1 & g2 >= 1 & g3 >= 1 & g4 >= 1)
```

```
  # Subsets
  data4 <- dat[check4, , drop = FALSE]
  data5 <- dat[check5, , drop = FALSE]

  # Unique gene counts (assuming geneID column is named "geneID")
  n_genes4 <- length(unique(data4$geneID))
  n_genes5 <- length(unique(data5$geneID))

  tibble(
    threshold     = thr,
    n_insertions4 = sum(check4),
    n_genes4      = n_genes4,
    n_insertions5 = sum(check5),
    n_genes5      = n_genes5
  )
}

# ------------------------------------------------------------
# 5. Run over all thresholds
# ------------------------------------------------------------
summary_thr <- bind_rows(lapply(thresholds, summarize_at_threshold))

print(summary_thr)

# Optionally save
write.table(
  summary_thr,
  file      = "./data.ta/threshold_sweep_summary.tsv",
  sep       = "\t",
  quote     = FALSE,
  row.names = FALSE
)

## ----------------------------------------------------------------
##  Add elbow-detection and pretty TIFF figure
## ----------------------------------------------------------------

library(ggplot2)
library(tidyr)
library(patchwork)

# ---------- 1) Elbow detection (Kneedle-style) ----------
detect_elbow <- function(x, y) {
  x_scaled <- (x - min(x)) / (max(x) - min(x))
  y_scaled <- (y - min(y)) / (max(y) - min(y))
  p1 <- c(x_scaled[1], y_scaled[1])
  p2 <- c(x_scaled[length(x_scaled)], y_scaled[length(y_scaled)])
  distances <- sapply(seq_along(x_scaled), function(i) {
    p <- c(x_scaled[i], y_scaled[i])
    abs(det(rbind(p2 - p1, p - p1))) / sqrt(sum((p2 - p1)^2))
  })
  which.max(distances)
}

idx_insert <- detect_elbow(summary_thr$threshold, summary_thr$n_insertions4)
```

```
idx_genes  <- detect_elbow(summary_thr$threshold, summary_thr$n_genes4)

elbow_insert <- summary_thr$threshold[idx_insert]
elbow_genes  <- summary_thr$threshold[idx_genes]

cat("Suggested elbow for insertions (4-group):", elbow_insert, "\n")
cat("Suggested elbow for genes (4-group)     :", elbow_genes,  "\n")

# ---------- 2) Long format for ggplot ----------
df_long_ins <- summary_thr %>%
  pivot_longer(cols = c("n_insertions4","n_insertions5"),
               names_to = "group",
               values_to = "n_insertions") %>%
  mutate(group = ifelse(group == "n_insertions4", "4-group", "5-group"))

df_long_genes <- summary_thr %>%
  pivot_longer(cols = c("n_genes4","n_genes5"),
               names_to = "group",
               values_to = "n_genes") %>%
  mutate(group = ifelse(group == "n_genes4", "4-group", "5-group"))

group_colors <- c("4-group" = "#1f78b4", "5-group" = "#e31a1c")

# ---------- 3) Insertions plot ----------
p_ins <- ggplot(df_long_ins,
                aes(x = threshold, y = n_insertions, color = group)) +
  geom_line(linewidth = 1) +
  geom_point(size = 3) +
  geom_vline(xintercept = elbow_insert, linetype = "dashed", color = "black") +
  annotate("label", x = elbow_insert,
           y = max(df_long_ins$n_insertions) * 0.9,
           label = paste("Elbow =", elbow_insert),
           size = 3, label.size = 0.2, fill = "white") +
  scale_color_manual(values = group_colors) +
  scale_x_continuous(breaks = summary_thr$threshold) +
  theme_bw(base_size = 13) +
  theme(
    legend.position = "top",
    legend.title = element_blank(),
    plot.title = element_text(face = "bold"),
    axis.text.x = element_text(angle = 45, hjust = 1)
  ) +
  labs(
    title = "TA insertions retained vs. count threshold",
    x = "Threshold (> X counts)",
    y = "Number of TA insertions"
  )

# ---------- 4) Genes plot (rotated x labels) ----------
p_genes <- ggplot(df_long_genes,
                  aes(x = threshold, y = n_genes, color = group)) +
  geom_line(linewidth = 1) +
  geom_point(size = 3) +
  geom_vline(xintercept = elbow_genes, linetype = "dashed", color = "black") +
  annotate("label", x = elbow_genes,
           y = max(df_long_genes$n_genes) * 0.925,
```

```r
        label = paste("Elbow =", elbow_genes),
        size = 3, label.size = 0.2, fill = "white") +
  scale_color_manual(values = group_colors) +
  scale_x_continuous(breaks = summary_thr$threshold) +
  theme_bw(base_size = 13) +
  theme(
    legend.position = "top",
    legend.title = element_blank(),
    plot.title = element_text(face = "bold"),
    axis.text.x = element_text(angle = 45, hjust = 1)
  ) +
  labs(
    title = "Unique genes represented vs. count threshold",
    x = "Threshold (> X counts)",
    y = "Number of genes"
  )

# ---------- 5) Combine and export ----------
combined_plot <- p_ins / p_genes +
  plot_layout(heights = c(1, 1)) +
  plot_annotation(
    title = "Threshold sensitivity analysis for pooled Tn-Seq selection",
    theme = theme(
      plot.title = element_text(face = "bold", hjust = 0.5, size = 16),
      legend.position = "top",
      legend.title = element_blank()
    )
  )

print(combined_plot)

# Save as TIFF (high resolution)
tiff("./data.ta/threshold_sweep_pretty.tiff",
     width = 8, height = 8, units = "in", res = 600)
print(combined_plot)
dev.off()

### Manuscript-friendly wording
### “Sensitivity analysis across dynamic count thresholds (100–5000)
### showed a sharp decline in gene representation beyond ~1000 reads.
### Therefore, a threshold of >500 reads was selected to balance library
### representativeness and robustness, with high-confidence insertions
### identified using a geometric mean score across the four pooling dimensions.”
```

TA insertions filtering

```
require(openxlsx)
require(dplyr)

# -----------------------------------------------------------------
# Parameters
# -----------------------------------------------------------------
thr        <- 500     # count threshold
max_gap_bp <- 50      # max distance (bp) between contiguous TA sites within same groups

# 1. Define the count columns exactly as in your dataset
count_cols <- c(
  # X pools
  "XA","XB","XC","XD","XE","XF","XG","XH",
  # Y pools
  paste0("Y", 1:12),
  # Z row pools
  "ZA","ZB","ZC","ZD","ZE","ZF","ZG","ZH",
  # Z column pools
  paste0("Z", 1:12)
)

# Convenience subsets
x_cols  <- c("XA","XB","XC","XD","XE","XF","XG","XH")
y_cols  <- paste0("Y", 1:12)
zl_cols <- c("ZA","ZB","ZC","ZD","ZE","ZF","ZG","ZH")
zn_cols <- paste0("Z", 1:12)

setwd("~/UF/Dental/rob/ana/")

### Reading dataset
#origin <- read.xlsx("./ta_insertions_annotated_all_samples.xlsx", sheet="Genes")
#colnames(origin)[c(2,3)] <- c("pos1","pos2")
#colnames(origin)[c(6,7)] <- c("start","end")

### Groups columns (for sanity check)
origin[1,11:18]
origin[1,19:30]
origin[1,31:38]
origin[1,39:50]

data.ta <- data.frame(origin, check01=0, check02=0)

### Loop for count 4 and 5
for ( i.row in 1:nrow(data.ta)) {

  if( length(which(data.ta[i.row,11:18]>thr))==1 &
      length(which(data.ta[i.row,19:30]>thr))==1 &
      length(which(data.ta[i.row,31:38]>thr))==1 &
      length(which(data.ta[i.row,39:50]>thr))==1 ) {
    data.ta[i.row,51] <- 1
  }

  g1 <- length(which(data.ta[i.row,11:18] > thr))  # Xletters
  g2 <- length(which(data.ta[i.row,19:30] > thr))  # Ynumbers
```

```
  g3 <- length(which(data.ta[i.row,31:38] > thr))  # Zletters
  g4 <- length(which(data.ta[i.row,39:50] > thr))  # Znumbers

  if( (g1 + g2 + g3 + g4) == 5  && g1 >= 1 && g2 >= 1 && g3 >= 1 && g4 >= 1 ) {
    data.ta[i.row,52] <- 1
  }
}

data.ta.4 <- data.ta[data.ta[,51]==1,]
data.ta.5 <- data.ta[data.ta[,52]==1,]

# ----------------------------------------------------------------
# Add relative position of TA within gene: (pos1 - start) / (end - start)
# ----------------------------------------------------------------
data.ta.4$rel_pos <- (data.ta.4$pos1 - data.ta.4$start) / (data.ta.4$end - data.ta.4$start)
data.ta.5$rel_pos <- (data.ta.5$pos1 - data.ta.5$start) / (data.ta.5$end - data.ta.5$start)

# Plate mapping: (Zletter, Znumber) -> plate_pos 1..96
z_rows <- c("A","B","C","D","E","F","G","H")
z_cols <- 1:12

plate_map <- expand.grid(
  Zletter = z_rows,
  Znumber = z_cols
)
# Ensure row-wise order: A1..A12, B1..B12, ..., H1..H12
plate_map <- plate_map[order(plate_map$Zletter, plate_map$Znumber), ]
plate_map$plate_pos <- seq_len(nrow(plate_map))  # 1..96

### FINAL FILTERING FOR 4 and 5

# + ------------------------------------------------------------------------------------------------ +
# Extract Zletter index (which of the 8 letter pools was >thr)
data.ta.4$Zletter <- z_rows[
  apply(data.ta.4[,31:38], 1, function(x) which(x > thr))
]
# Extract Znumber index (which of the 12 number pools was >thr)
data.ta.4$Znumber <- apply(data.ta.4[,39:50], 1, function(x) which(x > thr))

# NEW: Xlet/Ynum for 4-group (always exactly one hit per group by design)
data.ta.4$Xlet <- z_rows[
  apply(data.ta.4[,11:18], 1, function(x) which(x > thr))
]
data.ta.4$Ynum <- apply(data.ta.4[,19:30], 1, function(x) which(x > thr))

# ---- Add plate position using the mapping ----
data.ta.4 <- merge(
  data.ta.4,
  plate_map,
  by = c("Zletter", "Znumber"),
  all.x = TRUE
)

cols <- setdiff(names(data.ta.4), c("Zletter", "Znumber", "plate_pos"))
data.ta.4 <- data.ta.4[, c(cols, "Zletter", "Znumber", "plate_pos")]
data.ta.4 <- data.ta.4[with(data.ta.4, order(pos1)),]
```

```
### For each gene, pick the TA insertion with the largest total counts
data.ta.4.best <- data.ta.4 %>%
  mutate(
    total_reads = rowSums(across(all_of(count_cols)), na.rm = TRUE)
  ) %>%
  group_by(geneID) %>%
  slice_max(total_reads, n = 1, with_ties = FALSE) %>%  # one best TA per gene
  ungroup()

data.ta.4.best <- data.ta.4.best[with(data.ta.4.best, order(start)),]

# + ------------------------------------------------------------------------------------------------ +
# Helper for 5-group confidence: TRUE if group is ambiguous (top two hits too similar)
ambig_flag_fun <- function(x, thr) {
  idx <- which(x > thr)
  if (length(idx) <= 1) return(FALSE)   # 0 or 1 hit -> not ambiguous
  vals <- x[idx]
  if (length(vals) < 2) return(FALSE)
  vals <- sort(vals, decreasing = TRUE)
  ratio <- vals[1] / vals[2]
  # ambiguous if top two counts are too similar (ratio < 2)
  return(ratio < 2)
}

## ---- Decode Zletter for data.ta.5 ----
## 1 hit  > thr  -> use that one
## 2 hits > thr  -> keep the one with the larger count
data.ta.5$Zletter <- apply(data.ta.5[, 31:38], 1, function(x) {
  idx <- which(x > thr)

  if (length(idx) == 1) {
    return(z_rows[idx])
  } else if (length(idx) == 2) {
    # keep the one with the larger count
    idx_keep <- idx[which.max(x[idx])]
    return(z_rows[idx_keep])
  } else {
    return(NA_character_)  # safety fallback
  }
})

## ---- Decode Znumber for data.ta.5 ----
## same logic but we keep the numeric index 1..12
data.ta.5$Znumber <- apply(data.ta.5[, 39:50], 1, function(x) {
  idx <- which(x > thr)

  if (length(idx) == 1) {
    return(idx)
  } else if (length(idx) == 2) {
    idx_keep <- idx[which.max(x[idx])]
    return(idx_keep)
  } else {
    return(NA_integer_)    # safety fallback
  }
})
```

```
## ---- NEW: Xlet/Ynum for 5-group with tie-breaking ----
data.ta.5$Xlet <- apply(data.ta.5[,11:18], 1, function(x) {
  idx <- which(x > thr)
  if (length(idx) == 1) {
    z_rows[idx]
  } else if (length(idx) == 2) {
    idx_keep <- idx[which.max(x[idx])]
    z_rows[idx_keep]
  } else {
    NA_character_
  }
})

data.ta.5$Ynum <- apply(data.ta.5[,19:30], 1, function(x) {
  idx <- which(x > thr)
  if (length(idx) == 1) {
    idx
  } else if (length(idx) == 2) {
    idx_keep <- idx[which.max(x[idx])]
    idx_keep
  } else {
    NA_integer_
  }
})

## ---- NEW: Confidence metric + ambiguity flag for 5-group ----
ambig_X  <- apply(data.ta.5[,11:18],  1, ambig_flag_fun, thr = thr)
ambig_Y  <- apply(data.ta.5[,19:30],  1, ambig_flag_fun, thr = thr)
ambig_ZL <- apply(data.ta.5[,31:38],  1, ambig_flag_fun, thr = thr)
ambig_ZN <- apply(data.ta.5[,39:50],  1, ambig_flag_fun, thr = thr)

data.ta.5$ambiguous_5group <- ambig_X | ambig_Y | ambig_ZL | ambig_ZN

data.ta.5$conf_5group <- pmax(
  0,
  1 - 0.25 * (
    as.integer(ambig_X) +
      as.integer(ambig_Y) +
      as.integer(ambig_ZL) +
      as.integer(ambig_ZN)
  )
)

## ---- Add plate position using the mapping ----
data.ta.5 <- merge(
  data.ta.5,
  plate_map,
  by = c("Zletter", "Znumber"),
  all.x = TRUE
)

## ---- Put Zletter, Znumber, plate_pos at the end (like we did for data.ta.4) ----
cols <- setdiff(names(data.ta.5), c("Zletter", "Znumber", "plate_pos"))
data.ta.5 <- data.ta.5[, c(cols, "Zletter", "Znumber", "plate_pos")]
```

```
data.ta.5 <- data.ta.5[with(data.ta.5, order(pos1)),]

### For each gene, pick the TA insertion with the largest total counts
data.ta.5.best <- data.ta.5 %>%
  mutate(
    total_reads = rowSums(across(all_of(count_cols)), na.rm = TRUE)
  ) %>%
  group_by(geneID) %>%
  slice_max(total_reads, n = 1, with_ties = FALSE) %>%  # one best TA per gene
  ungroup()

data.ta.5.best <- data.ta.5.best[with(data.ta.5.best, order(start)),]

# ------------------------------------------------------------------
# NEW: Find contiguous cross-gene TA clusters within same (Xlet,Ynum,Zletter,Znumber)
# starting from data.ta.4 and data.ta.5
# ------------------------------------------------------------------
find_neighbor_insertions <- function(df, max_gap = 50) {
  # df must contain: insertion, geneID, pos1, Xlet, Ynum, Zletter, Znumber

  df_work <- df[order(df$Xlet, df$Ynum, df$Zletter, df$Znumber, df$pos1), ]

  if (nrow(df_work) < 2) return(character(0))

  # group by plate well (Xlet,Ynum,Zletter,Znumber)
  grp <- interaction(df_work$Xlet, df_work$Ynum,
                     df_work$Zletter, df_work$Znumber,
                     drop = TRUE)

  keep_idx <- logical(nrow(df_work))

  for (g in split(seq_len(nrow(df_work)), grp)) {
    if (length(g) < 2) next

    # walk through this group to build contiguous clusters
    current_cluster <- g[1]
    for (k in 2:length(g)) {
      i_prev <- g[k-1]
      i_curr <- g[k]
      if (df_work$pos1[i_curr] - df_work$pos1[i_prev] <= max_gap) {
        # still contiguous in genomic space
        current_cluster <- c(current_cluster, i_curr)
      } else {
        # cluster ended; check if cross-gene
        if (length(unique(df_work$geneID[current_cluster])) > 1) {
          keep_idx[current_cluster] <- TRUE
        }
        current_cluster <- g[k]
      }
    }
    # last cluster in this group
    if (length(unique(df_work$geneID[current_cluster])) > 1) {
      keep_idx[current_cluster] <- TRUE
    }
  }
```

```
  df_work$insertion[keep_idx]
}

neighbors_ins4 <- find_neighbor_insertions(data.ta.4, max_gap = max_gap_bp)
neighbors_ins5 <- find_neighbor_insertions(data.ta.5, max_gap = max_gap_bp)

# + ------------------------------------------------------------------------------------------------ +
### EXCEL WRITING (reorder only at export so rel_pos is column 11)
# + ------------------------------------------------------------------------------------------------ +

# Front columns: rel_pos immediately after annotation (col 11)
front10 <- c("insertion","pos1","pos2","geneID","name",
             "start","end","length","strand","annotation")
front_with_rel <- c(front10, "rel_pos")

# Build clean export data frames, dropping check01/check02/total_reads
df4      <- subset(data.ta.4,      select = -c(check01, check02))
df4_best <- subset(data.ta.4.best, select = -c(check01, check02, total_reads))
df5      <- subset(data.ta.5,      select = -c(check01, check02))
df5_best <- subset(data.ta.5.best, select = -c(check01, check02, total_reads))

reorder_for_export <- function(df) {
  keep_front <- front_with_rel[front_with_rel %in% names(df)]

  # tail block: plate-related + confidence columns, in this order if present
  tail_cols <- c(
    "Xlet","Ynum","Zletter","Znumber","plate_pos",
    "ambiguous_5group","conf_5group"
  )
  tail_cols <- tail_cols[tail_cols %in% names(df)]

  # everything else (non-front, non-count, non-tail)
  middle <- setdiff(names(df), c(keep_front, count_cols, tail_cols))

  df[, c(keep_front, count_cols, middle, tail_cols)]
}

df4      <- reorder_for_export(df4)
df4_best <- reorder_for_export(df4_best)
df5      <- reorder_for_export(df5)
df5_best <- reorder_for_export(df5_best)

# Neighbor tables: same columns as df4 / df5, just subset by insertion ID
neighbors4 <- df4[df4$insertion %in% neighbors_ins4, ]
neighbors5 <- df5[df5$insertion %in% neighbors_ins5, ]

# Create a blank workbook
OUT <- createWorkbook()
# Add some sheets to the workbook
addWorksheet(OUT, "4 across groups")
addWorksheet(OUT, "best 4 for each gene")
addWorksheet(OUT, "5 across groups")
addWorksheet(OUT, "best 5 for each gene")
addWorksheet(OUT, "neighbors 4")
addWorksheet(OUT, "neighbors 5")
```

```
# Write the data to the sheets
writeData(OUT, sheet="4 across groups",      x = df4)
writeData(OUT, sheet="best 4 for each gene", x = df4_best)
writeData(OUT, sheet="5 across groups",      x = df5)
writeData(OUT, sheet="best 5 for each gene", x = df5_best)
writeData(OUT, sheet="neighbors 4",          x = neighbors4)
writeData(OUT, sheet="neighbors 5",          x = neighbors5)

# Setting columns width (first up to 55 columns)
for (nm in c("4 across groups","best 4 for each gene",
             "5 across groups","best 5 for each gene",
             "neighbors 4","neighbors 5")) {
  df_name <- switch(
    nm,
    "4 across groups"       = "df4",
    "best 4 for each gene"  = "df4_best",
    "5 across groups"       = "df5",
    "best 5 for each gene"  = "df5_best",
    "neighbors 4"           = "neighbors4",
    "neighbors 5"           = "neighbors5"
  )
  df <- get(df_name)
  ncols <- ncol(df)
  max_cols <- min(55, ncols)

  # base widths: default 7
  widths <- rep(7, max_cols)
  if (max_cols >= 1)  widths[1]  <- 14          # insertion
  if (max_cols >= 2)  widths[2:9] <- 9          # pos1,pos2,geneID,name,start,end,length,strand
  if (max_cols >= 10) widths[10] <- 75          # annotation
  if (max_cols >= 11) widths[11] <- 9           # rel_pos

  # plate + confidence columns get width 8 if within first max_cols
  tail_for_width <- c("Xlet","Ynum","Zletter","Znumber","plate_pos",
                      "ambiguous_5group","conf_5group")
  idx_tail <- match(tail_for_width, names(df))
  idx_tail <- idx_tail[!is.na(idx_tail) & idx_tail <= max_cols]
  if (length(idx_tail) > 0) {
    widths[idx_tail] <- 8
  }

  setColWidths(
    OUT,
    sheet  = nm,
    cols   = 1:max_cols,
    widths = widths
  )
}

# Reorder worksheets (keep main four first, then neighbors)
worksheetOrder(OUT) <- c(1,2,3,4,5,6)

### Conditional Formatting: counts > thr
blueStyle <- createStyle(bgFill = "#80D1FF")

df_list <- list(
```

```
  "4 across groups"      = df4,
  "best 4 for each gene" = df4_best,
  "5 across groups"      = df5,
  "best 5 for each gene" = df5_best,
  "neighbors 4"          = neighbors4,
  "neighbors 5"          = neighbors5
)

# Center both horizontally and vertically
centerHV <- createStyle(
  halign = "center",
  valign = "center"
)
# Left horizontal only (used to override column 10)
centerH <- createStyle(
  halign = "left"
)

for (sheet in names(df_list)) {
  df <- df_list[[sheet]]
  # count columns by name
  idx_counts <- match(count_cols, names(df))
  idx_counts <- idx_counts[!is.na(idx_counts)]

  if (length(idx_counts) > 0) {
    conditionalFormatting(
      OUT,
      sheet = sheet,
      cols  = idx_counts,
      rows  = 2:(nrow(df) + 1),
      type  = "expression",
      style = blueStyle,
      rule  = paste0(">", thr)
    )
  }

  ### Center all
  addStyle(
    OUT,
    sheet = sheet,
    style = centerHV,
    rows = 1:(nrow(df)+1),   # include header row
    cols = 1:ncol(df),
    gridExpand = TRUE,
    stack = TRUE
  )
  ### Recenter column 10 (left horizontal)
  addStyle(
    OUT,
    sheet = sheet,
    style = centerH,
    rows = 1:(nrow(df)+1),   # include header row
    cols = 10,
    gridExpand = TRUE,
    stack = TRUE
  )
```

```
  ### Conditional Formatting for the relative position of the TA insertion
  conditionalFormatting(
    OUT,
    sheet = sheet,
    cols = c(11),
    rows = 2:(nrow(df)+1),  # exclude header row
    type = "colorScale",
    style = c("green", "yellow", "red"),
    rule = quantile(df[,11], c(0, 0.5, 1), na.rm = TRUE)  # explicitly set scale values
  )
}

# + ----------------------------------------------------------------------------------------------------- +
### Define color of the groups columns (X, Y, Z-letter, Z-number)
# + ----------------------------------------------------------------------------------------------------- +
header_colors <- list(
  X  = "#DAEEF4",
  Y  = "#B7DEE8",
  ZL = "#FDE9D9",
  ZN = "#FBD5B4"
)

for (sheet in names(df_list)) {
  df <- df_list[[sheet]]

  x_idx  <- match(x_cols,  names(df)); x_idx  <- x_idx[!is.na(x_idx)]
  y_idx  <- match(y_cols,  names(df)); y_idx  <- y_idx[!is.na(y_idx)]
  zl_idx <- match(zl_cols, names(df)); zl_idx <- zl_idx[!is.na(zl_idx)]
  zn_idx <- match(zn_cols, names(df)); zn_idx <- zn_idx[!is.na(zn_idx)]

  if (length(x_idx) > 0) {
    addStyle(OUT, sheet = sheet,
             style = createStyle(fgFill = header_colors$X),
             rows = 1, cols = min(x_idx):max(x_idx),
             gridExpand = TRUE, stack = TRUE)
  }
  if (length(y_idx) > 0) {
    addStyle(OUT, sheet = sheet,
             style = createStyle(fgFill = header_colors$Y),
             rows = 1, cols = min(y_idx):max(y_idx),
             gridExpand = TRUE, stack = TRUE)
  }
  if (length(zl_idx) > 0) {
    addStyle(OUT, sheet = sheet,
             style = createStyle(fgFill = header_colors$ZL),
             rows = 1, cols = min(zl_idx):max(zl_idx),
             gridExpand = TRUE, stack = TRUE)
  }
  if (length(zn_idx) > 0) {
    addStyle(OUT, sheet = sheet,
             style = createStyle(fgFill = header_colors$ZN),
             rows = 1, cols = min(zn_idx):max(zn_idx),
             gridExpand = TRUE, stack = TRUE)
  }
}
```

```
# + ------------------------------------------------------------------------------------------------------ +
### Define color of the plate-related columns (rel_pos, Xlet, Ynum, Zletter, Znumber, plate_pos, ambig/conf)
# + ------------------------------------------------------------------------------------------------------ +
greenStyle <- createStyle(fgFill = "#DAE4C0")
plate_cols <- c("rel_pos","Xlet","Ynum","Zletter","Znumber","plate_pos")

for (sheet in names(df_list)) {
  df <- df_list[[sheet]]
  p_idx <- match(plate_cols, names(df))
  p_idx <- p_idx[!is.na(p_idx)]

  if (length(p_idx) > 0) {
    addStyle(
      OUT,
      sheet = sheet,
      style = greenStyle,
      rows = 1,
      cols = p_idx,
      gridExpand = TRUE,
      stack = TRUE
    )
  }
  # For 5-group-related sheets, also shade ambiguous/conf columns (wherever they are)
  if (sheet %in% c("5 across groups","best 5 for each gene","neighbors 5")) {
    extra_idx <- match(c("ambiguous_5group","conf_5group"), names(df))
    extra_idx <- extra_idx[!is.na(extra_idx)]
    if (length(extra_idx) > 0) {
      addStyle(
        OUT,
        sheet = sheet,
        style = greenStyle,
        rows = 1,
        cols = extra_idx,
        gridExpand = TRUE,
        stack = TRUE
      )
    }
  }
}

# Export the file
saveWorkbook(OUT,
             paste0("./data.ta/ta_insertions_annotated_all_samples_filtered_",
                    thr,"_v4_neighbors.xlsx"),
             overwrite=TRUE)
```

References

1. McLellan LK, Anderson ME, Grossman AD. 2022. TnSmu1 is a functional integrative and conjugative element in *Streptococcus mutans* that when expressed causes growth arrest of host bacteria. Mol Microbiol 118:652–669.
